# Quantitative Spatiotemporal Analysis of Intracellular Kinase Activity in Metastatic Breast Cancer Cells Using a Microfluidic-Based Lateral Diffusion Assay

**DOI:** 10.1021/acs.analchem.6c01060

**Published:** 2026-06-21

**Authors:** Brendan T. Fuller, Travis H. Jones, Emily T. Chan, Malcolm W. D’Souza, Kathryn E. Luker, Gary D. Luker, Jonathan W. Song

**Affiliations:** † Department of Biomedical Engineering, 2647 The Ohio State University, Columbus, Ohio 43210, United States; ‡ Department of Mechanical and Aerospace Engineering, The Ohio State University, Columbus, Ohio 43210, United States; § Interdisciplinary Biophysics Graduate Program, The Ohio State University, Columbus, Ohio 43210, United States; ∥ Department of Physics, The Ohio State University, Columbus, Ohio 43210, United States; ⊥ Biomedical Science, College of Medicine, The Ohio State University, Columbus, Ohio 43210, United States; # Department of Radiology, 1259 University of Michigan, Ann Arbor, Michigan 48109, United States; ∇ Biointerfaces Institute, University of Michigan, Ann Arbor, Michigan 48109, United States; ○ Department of Biomedical Engineering, University of Michigan, Ann Arbor, Michigan 48109, United States; ◆ Comprehensive Cancer Center, The Ohio State University, Columbus, Ohio 43210, United States

## Abstract

Mass transport by diffusion helps shape extracellular gradients of soluble signaling molecules in tumor microenvironments and other physiological settings. Microfluidic technologies are conducive to generating predictable chemical gradients. Yet, they often require specialized fluid-handling expertise and external pumping systems. We designed and implemented a simple microfluidic-based lateral diffusion assay (LDA) that enables reproducible and predictable biomolecular gradients without pumps and is devoid of any confounding pressure-driven flow. Using breast cancer cells that coexpress a kinase translocation reporter (KTR) for Akt, we demonstrate quantitative, real-time analysis of intracellular kinase signaling in response to diffusion-limited extracellular gradients of epidermal growth factor (EGF) in the LDA. We observed temporally and spatially staggered Akt activation and deactivation in KTR cells, with these signaling dynamics correlating to the rate of EGF delivery across zonal boundaries or EGF flux. We identified a threshold EGF concentration required for Akt activation in the median cell population and showed that this threshold concentration increases with cell density. Using mathematical modeling that incorporated empirically derived parameters, we accurately predicted individual cell Akt activation patterns among different EGF source concentrations and cell densities. Finally, we showed that both activation and deactivation patterns depend on the rate of the EGF concentration change, revealing the pivotal role of EGF flux in controlling signaling dynamics. Together, these findings establish the LDA as a powerful and accessible platform for dissecting the dynamics of extracellular gradients in controlling intracellular signaling.

## Graphical abstract

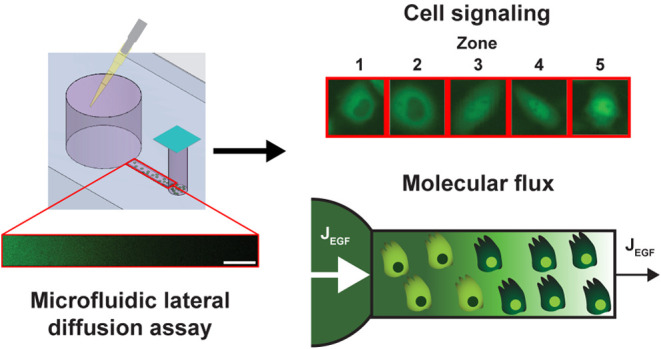

## Introduction

Intracellular kinases such as PI3K and Akt are critical regulators of cancer cell growth, proliferation, and migration.^1^ Dysregulation of kinase signaling pathways is common in highly aggressive triple-negative breast cancer (TNBC) and other malignancies.^2–4^ Activators of intracellular kinases in cancer cells include signaling biomolecules secreted by cancer and noncancer cells, such as fibroblasts and immune cells.^5–7^ This biomolecular environment in tumors is shaped into spatial gradients by transport phenomena of diffusion (transport due to concentration differences) and convection (transport due to bulk fluid motion due to pressure differences). Biomolecular gradients in tumors help drive aggressive traits of cancer cells, such as proliferation, invasion, and stemness.^8^ Extrinsic factors such as biomolecular gradients and the engaged signaling pathways also vary dynamically as tumors progress and adapt to therapy.^9,10^ Thus, unraveling the interplay between spatial biomolecular changes in the tumor microenvironment (TME) and cancer cell signaling is essential for advancing our mechanistic understanding of tumor progression and for developing more effective cancer treatments.^11,12^

The TME dynamically coevolves with cancer cells, but conventional approaches for understanding intracellular kinase activity, such as qPCR, ELISA, and Western blotting, analyze fixed samples and therefore provide end point information. Conversely, kinase translocation reporter (KTR) constructs stably expressed in cells enable real-time visualization and quantification of intracellular kinase activity.^13,14^ Prior studies have used KTRs in cancer cells to show temporal dynamics and heterogeneity in Akt and ERK signaling.^15–17^ However, to date, quantitative, single, live-cell imaging technology has not been used to help resolve the impact of extracellular biomolecular gradients on intracellular Akt or other kinase signaling. Furthermore, physical forces such as shear stresses induced by convective flow directly affect intracellular kinase signaling, making it essential to minimize convective flows when developing biomolecular gradients.^18,19^ Integrating KTR technology with microfluidic systems with controlled, diffusion-based gradients would allow elucidation of the dynamics of spatially dependent, intracellular signaling with live-cell imaging.

Microfluidic-based models have been widely used for studying the biological effects of controllable biomolecular gradients, most prominently directed cell migration.^20–22^ However, the approaches for generating gradients in microfluidic models are often constrained by complex channel designs and nonuser friendly and active perfusion pumps and apparatus.^23–26^ Microfluidic gradient assays have a low adoption rate by biologists, and the requisite microsystems engineering and fluid-handling expertise may be an important contributing factor to this status.^27^ Furthermore, while microfluidic systems are largely considered diffusion-limited cell microenvironments, even small differences in hydrostatic pressure between the inlet and outlet of a microchannel can impart confounding convective flows.^23,28^ Therefore, it can be challenging to isolate the effects of diffusion from convection within the same microchannel. Recent studies have used KTRs in microfluidic devices to show how pulsed delivery of extracellular stimuli at different rates alters signaling dynamics of ERK.^29,30^ However, this system still relies on external pumps. Thus, there is an unmet need for simple and pumpless microchannel systems that are devoid of any convection (or “convection neutral”), which will enable real-time visualization and quantification of intracellular Akt activity due to extracellular and diffusion-limited spatial gradients.

To address this gap, we have developed a lateral diffusion assay (LDA)—a simple, occluded-outlet microfluidic device that generates diffusion-driven gradients from a single pipetted droplet containing reagents of known starting concentrations. In microchannel-based assays, pipetting of even small volumes of liquid (<10 μL) imparts convective flow-inducing pressure and momentum. Hence, isolating the effects on cell responses that are conferred by diffusion of signaling molecules only and in the absence of confounding convective flow is challenging within a microfluidic system. Our design eliminated convective flow and enabled us to generate precisely controlled spatial gradients of EGF and the rate of EGF delivery (or flux of EGF) to measure Akt activation via a KTR in TNBC cells. We determined the EGF threshold concentration needed to activate the Akt and found that higher cell densities required higher concentrations of EGF for Akt activation. Interestingly, we found differences in Akt activation and deactivation patterns depending on the starting EGF concentration and cell density. Notably, these results correlated to the flux of EGF in each zone. In addition, we tested the effects of clathrin-mediated endocytosis and observed an increase in the magnitude of Akt signaling. These findings highlight the integration of LDA with KTR cells as a novel technology for profiling spatiotemporal Akt signaling under controlled diffusion.

## Results

### Development of a Controllable Lateral Diffusion Assay Using a Convection-Neutral Microfluidic Channel with an Occluded Outlet

Key features of the lateral diffusion assay (LDA) are an interconnected large inlet port (6 mm diameter), a microchannel (4 mm × 0.15 mm × 0.5 mm, length × height × width), and a small outlet port (1.5 mm diameter) (Figure 1A). Since the length of microchannel is much greater than the height and width, we approximated mass transport to be 1-D only. Prior to commencing experiments, the outlet of the LDA was fully occluded with microfluidic tape. This occlusion blocked pressure-driven convection within the microchannel such that transport of solutes occurs solely by diffusion (Figure 1B). Experiments were prepared using a three-day workflow prior to cell imaging (Figure 1B, details provided in Materials and Methods section). The diffusion properties in the LDA were characterized with 10 kDa fluorescein isothiocyanate (FITC)-dextran as a surrogate fluorescent tracer for epidermal growth factor (EGF), which has a molecular weight of ∼6 kDa.^31^ EGF is our signaling molecule of interest as it is the ligand for EGF receptor (EGFR) and upstream of Akt signaling. EGFR is also overexpressed in the MDA-MB-231 cells used in this study.^32^

**Figure 1:**
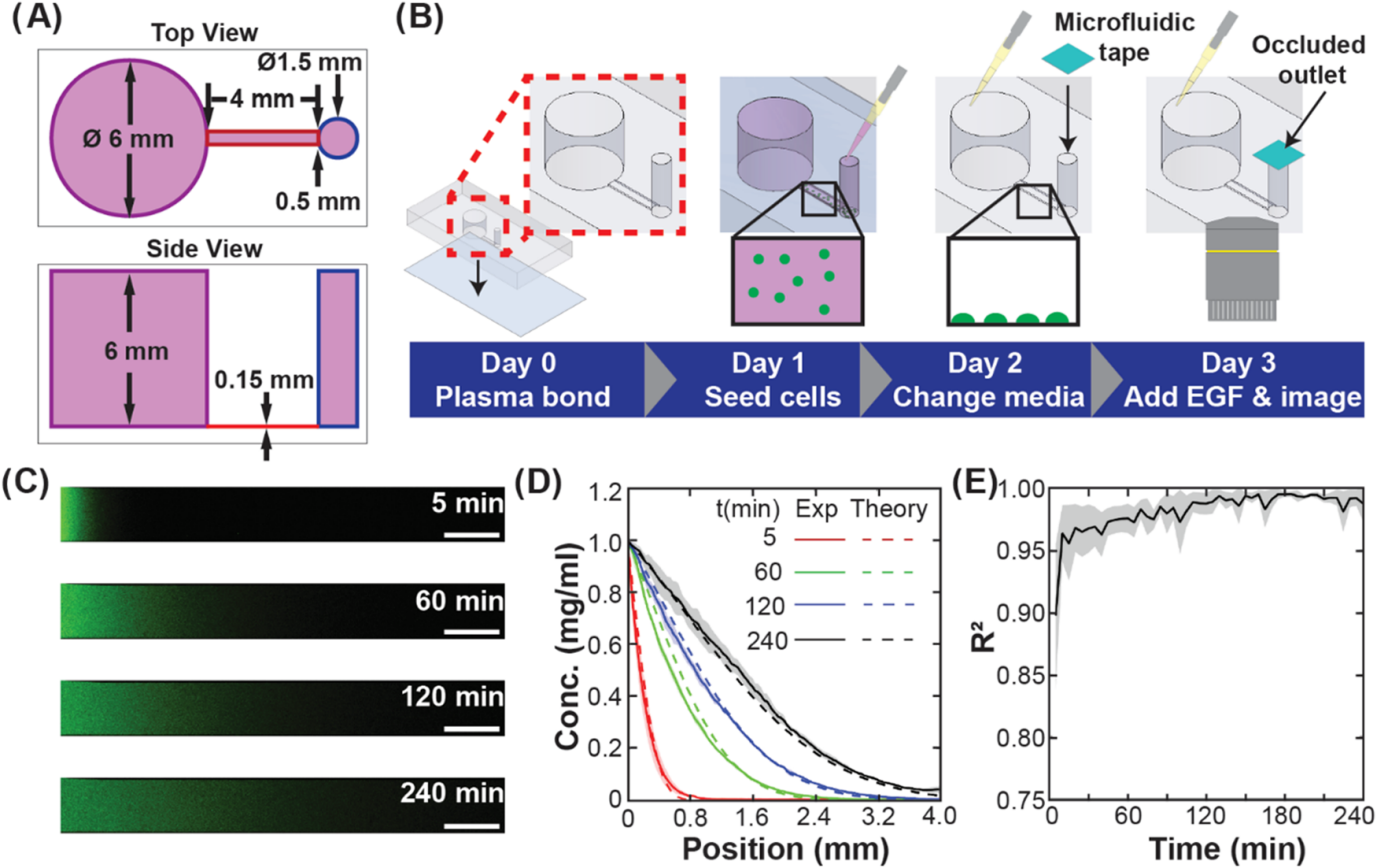
Microfluidic lateral diffusion assay (LDA). (A) Schematic of the LDA with connected inlet (purple), microchannel (red), and occluded outlet (blue) to eliminate convection. All features are drawn proportionally. (B) Workflow of the LDA for seeding fluorescently labeled cells **(day 1)**, media exchange **(day 2)**, and EGF introduction **(day 3)** done with simple pipetting. Imaging was done on **day 3** in the optically transparent LDA. (C) Representative images profiling the diffusion of 10 kDa FITC-dextran. Timestamps indicate the time elapsed after the introduction of the fluorescent tracer in the inlet of the LDA (Scale bar = 500 μm). (D) Comparison of experimental concentration in the LDA and theoretical concentration profiles using the forward Euler method. Solid lines are average concentration in the channel across experiments (*n* = 4). Shaded region represents ± one standard deviation. Dashed line is the theoretical concentration profile of 10 kDa FITC-dextran. (E) Average *R* ^2^ values across *n* = 4 experiments confirm strong agreement between experimental and theoretical diffusion, validating diffusion-dominant transport. Shaded region represents ± one standard deviation.

We first imaged the diffusion of 10 kDa FITC-dextran in an acellular LDA setup over 240 min, with images acquired every 5 min (Figure 1C). FITC-dextran was added as a concentrated solution (10 mg/mL) to the inlet port of the LDA prefilled with phosphate-buffered saline to yield an approximate initial concentration of 1 mg/mL. However, the process led to a time dependence of source concentration (Figure S1) and a nonhomogenous boundary condition, which necessitated a numerical approximation to solve the 1-D diffusion profile rather than a simple analytical solution for a fixed source (eq 7, Materials and Methods). Using a calibration curve of known 10 kDa FITC-dextran concentrations, we measured the concentration of dye in the channel at discrete locations and times. Furthermore, the experimentally observed and numerically calculated concentrations show strong overlap (Figure 1D) with average *R* ^2^ > 0.95 (Figure 1E). The initial lower *R* ^2^ values can be accounted for by the sharp diffusion profile at early time points resulting in small total sum of squares values and therefore sensitivity to synchronizing model and experiment time. This sensitivity dissipates as the time scales become large compared to the error in synchronizing data. The concentration gradient approached a linear profile by the 240 min time point (Figure 1D). As a further benchmark, we estimated the diffusion coefficient of the 10 kDa FITC-dextran that would minimize the sum of squares error of each time point for all runs. The average estimated diffusion coefficient across all four devices was 86.03 ± 4.32 μm^2^/s which equates to a relative standard deviation of 5% showing good repeatability. There was a −6 μm^2^/s difference between measured and theoretical diffusion (93.67 μm^2^/s). This could be due to a negligible counter flow (−0.4 μm/min), or alternatively, a slightly higher hydrodynamic radius due to aggregation of the dextran. Regardless, the Peclet number over the characteristic length of 1152 μm ( $L=\sqrt{D_{theory}t}$ ) is 0.082, which corresponds with a diffusion-dominant transport. The agreement of the theoretical and experimental data affirms that diffusion is the primary mechanism of mass transport in our microchannel with convection being negligible.

### Extracellular Biomolecular Gradient of EGF Controls Spatiotemporal Activation of Intracellular Akt

Next, we assessed intracellular Akt activation prompted by the controlled diffusion of extracellular EGF gradients in the LDA. We monitored intracellular Akt activation using timelapse microscopy of MDA-MB-231 human breast cancer cells stably expressing KTR constructs for Akt, hereafter referred to as simply KTR cells^13^ (Figure 2A). The KTR cells were seeded in the LDA, and Akt activity was confirmed by the relative average fluorescence intensity between the cytoplasm and nucleus, hereon referred to as the cytoplasmic-to-nuclear ratio (CNR). We considered a cell with a CNR > 1 to be in an active Akt state (see Materials and Methods). Time between seeding cells in the device and finishing imaging was <72 h. Importantly, we observed 100% viability after 72 h of culture time in the LDA (Figure S2A). As a positive control, we heat-treated cells at 65 °C for 2 min. Heat-treated cells showed almost complete cell membrane disruption (Figure S2B). Along the length of the LDA, we established five congruent zones containing a subpopulation of the KTR cells, with each zone being 800 μm long (Figure 2B). The CNR_*i*_ represents the average CNR of the subpopulation of cells within **Zone** ***i*** which was averaged at each time point (5 min).

**Figure 2:**
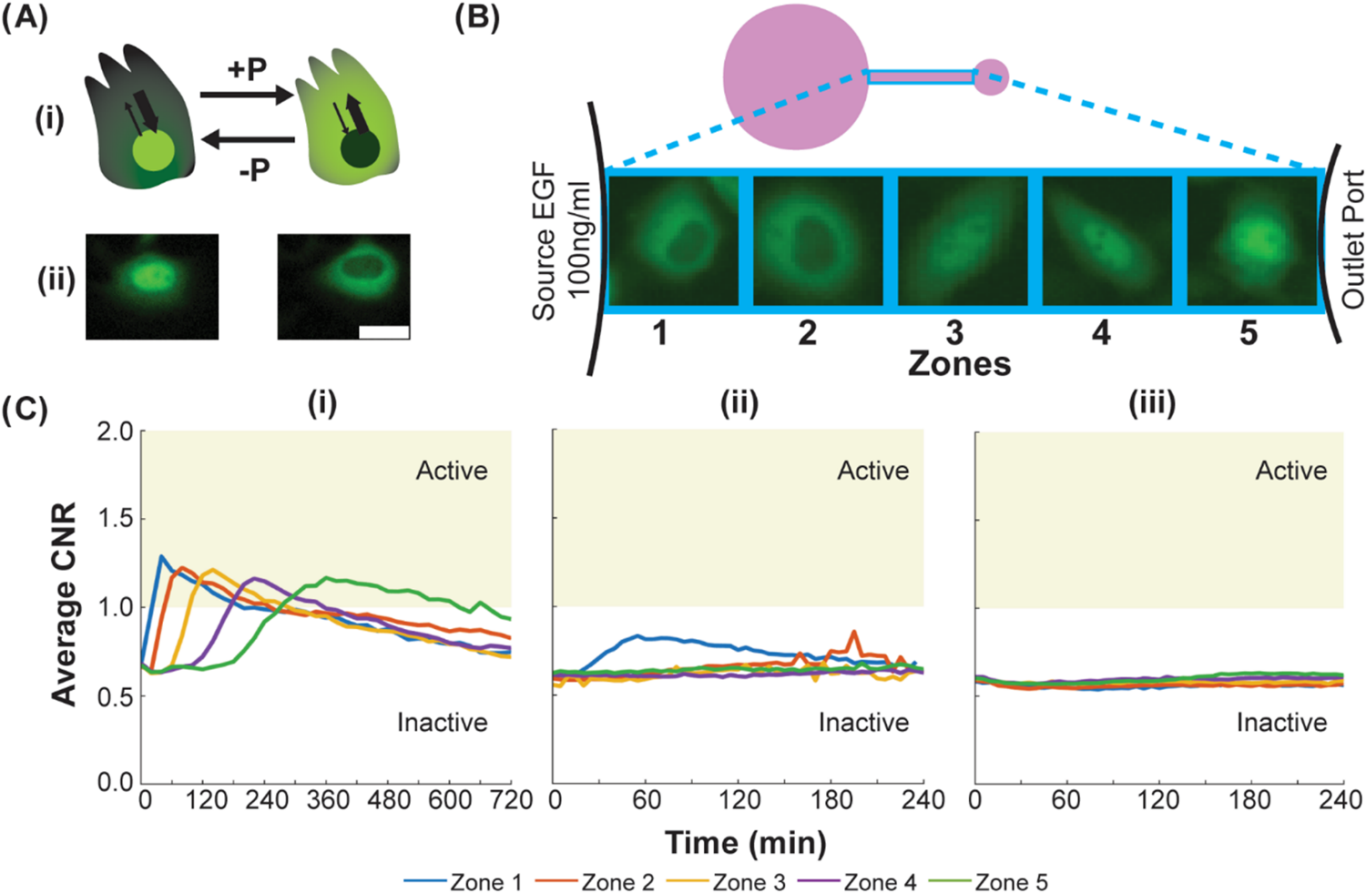
Exogenous EGF gradients in the LDA create spatially distinct zones of intracellular Akt activation in TNBC cells. (A) (i) KTR shuttles between the nucleus and cytoplasm due to phosphorylation (P) to indicate inactive (left) or active (right) Akt, respectively. (ii) Live-cell fluorescent images of an inactive (left) and active (right) KTR (scale bar = 25 μm). (B) Division of the LDA into five congruent zones with a representative cell from each zone after 1 h of stimulation by an EGF gradient. (C) Time-course plots of CNR_*i*_ for each zone. (i), Activation under 100 ng/mL EGF source concentration produces spatially dependent activation zones. Peak activation time for Zone 1 −40 min; Zone 2 −80 min; Zone 3 −140 min; Zone 4 −220 min; Zone 5 −360 min. The average standard deviation of all CNR over the first 240 min was 0.225. (ii), Control with low serum media (0.1% FBS and no EGF). The average standard deviation was 0.073. (iii), Co-treatment with 100 ng/mL EGF and 10 μM of EGFR inhibitor erlotinib. The average standard deviation was 0.058.

We added 100 ng/mL of EGF to the inlet port of the LDA, which resulted in a spatiotemporally staggered activation of the KTR cells. Specifically, the subpopulation of cells in each zone reached its maximum CNR_*i*_ (or “peak activation”) at different time points and sequentially from **Zone 1** to **Zone 5** (Figure 2C­(i)). Moreover, the spatial and temporal activation patterns of the KTR cells in the LDA obeyed a diffusion-like response (Figure S3) in agreement with our FITC-dextran diffusion measurements (Figure 1). For the control condition (0.1% FBS and no EGF), the CNR_*i*_ remained consistently below one, thereby suggesting no considerable off-target activation in the LDA (Figure 2C­(ii)). The small increase in CNR_1_ is likely due to the introduction of fresh media. We also coapplied EGF (100 ng/mL) with Erlotinib (10 μM, MW = 0.39 kDa), which is a small-molecule inhibitor that blocks the intracellular phosphorylation site of EGFR.^33^ When EGF and Erlotinib were added simultaneously to the source inlet of the LDA, Akt activation downstream of EGFR in the KTR cells was abrogated (Figure 2C­(iii)). Erlotinib is ∼15× less in MW and correspondingly has a much smaller hydrodynamic radius than EGF. These physical properties of Erlotinib may enhance its potency in the diffusion-limited setting of the LDA in inhibiting intracellular signaling downstream of EGF. Unlike the EGF-treated conditions, which were conducted for 720 min (Figure 2C­(i)), the control experiments and Erlotinib-treated conditions were concluded at 240 min as we observed no significant changes in CNRs (Figure 2C­(ii) and (iii)).

### The Flux of EGF Determines Zonal-Dependent Activation and Deactivation Signaling Dynamics of Akt

In the LDA, solute concentrations change more rapidly near the inlet source and are time-dependent (Figure 1D). These observations prompted us to identify the effects of the rate of EGF introduction into each zone, or diffusive flux (henceforth referred to simply as “flux”) of EGF across a known boundary (e.g., transition between zones in the LDA), on Akt signaling in KTR cell subpopulations. The flux of EGF is governed by Fick’s first law in 1-D:

1 $$J_{\mathrm{EGF}}=-D\frac{\partial c}{\partial x}$$

where *J*_EGF_ is the flux of EGF in the LDA, *D* is the diffusion coefficient (∼1.6 × 10^–10^ m^2^/s), and *∂c*/*∂x* is the concentration gradient through the channel. Using the forward Euler method to solve Fick’s Second Law, we showed zonal differences in EGF concentration changes over time (Figure 3C).

**Figure 3:**
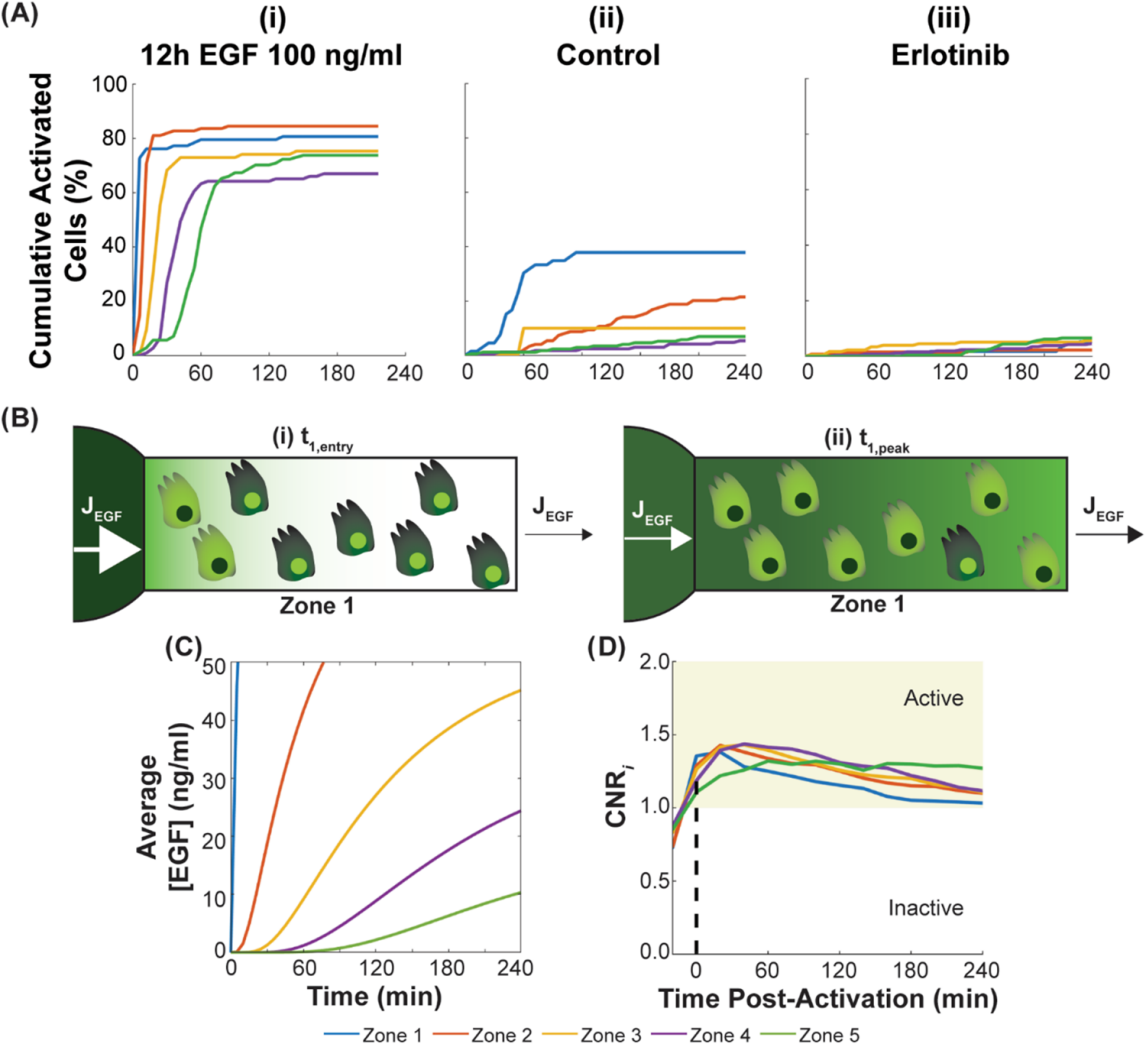
Effects of EGF fluctuations on signaling patterns of KTR cells. (A) Cumulative percentage of cells in each zone that reached activation levels (CNR > 1) for (i) 100 ng/mL EGF, (ii) no EGF, and (iii) 100 ng/mL EGF and 10 μM Erlotinib. (B) Illustration of Zone 1 for *t*_1,entry_ and *t*_1,peak_. (i) The time of entry of the minimum concentration of EGF required to activate a KTR cell is *t*_i,entry_. (ii) The time to reach the maximum average CNR in a zone is *t*_*i*,peak_. (C) EGF concentration plotted over time for each zone. This graph is for a source concentration of 100 ng/mL of EGF. (D) Activation times are synced to start at the same time point for all cells (black dashed line), and CNRs are then averaged for activating cells in each zone. Number of cells tracked in Zone 1–5 are *n* = 61, 73, 81, 80, and 93, respectively.

While all zones in the EGF condition exceeded the minimum amount of EGF needed for Akt activation, we observed an inverse relationship between the percentage of activated KTR cells within each zone and the distance away from the EGF inlet source (Figure 3A). **Zones 1 and 2** of the EGF condition had the highest percentages of activated cells at 80.6 and 84.5%, respectively. **Zones 4 and 5** had the lowest percentages of activated cells at 67.0 and 73.8%, respectively. There was a similar trend in the control condition but with much lower percent activation, again indicating that the introduction of fresh media was enough to activate a small number of cells in **Zones 1** and **2**. There was no observable trend in the erlotinib condition with activation levels near zero throughout the device (Figure 3A, Table S3). Collectively, these results suggest that a higher percentage, or more uniform, activation of KTR cells is achieved in the zone with the largest *J*_EGF_ (eq 1) observed in the LDA (Figure 3A­(i)). Moreover, the outcome for **Zones 1** and **2** of nearly complete but not total (or 100%) activation of the population of KTR cells in response to EGF is in accordance with previous observations in both microfluidic and well plate settings.^17^

Next, for each zone, we assessed the effect of *J*_EGF_ on peak activation (maximum CNR_*i*_) time (*t*_*i*_,_peak_). We hypothesized that there was a time delay for each zone (φ_*i*_,_delay_) that related to *J*_EGF_ and was the reason for the reduction in peak CNR_*i*_ in the latter zones. To quantify φ_*i*_,_delay_, we defined the time for a zone to reach a minimum EGF concentration that would enable activation in the KTR cell subpopulation (*t*_*i*_,_entry_) (Figure 3B). The minimum EGF concentration was chosen based on serial dilution experiments in 24-well plates and was observed to be ∼1 ng/mL of EGF. eq 7 was then used to calculate a t_*i*_,_entry_ for each zone of the LDA (Table S2). A φ_*i*_,_delay_ was then calculated for each zone by taking the difference between *t*_*i*_,_peak_ and *t*_*i*_,_entry_:

2 $$\varphi_{i,\mathrm{delay}}=t_{i,\mathrm{peak}}-t_{i,\mathrm{entry}}$$

where φ_*i*_,_delay_ increased as the distance from the EGF source increased (Table S2). Moreover, we quantified the peak, net *J*_EGF_ for each zone, and found that it was inversely correlated with an increasing φ_*i*_,_delay_. Hence, as the rate of EGF introduction for each zone diminished, the time to reach peak CNR_*i*_ increased (Figure 3C).

We speculated that averaging CNRs relative to a global *t* = 0 may lower the magnitude of peak activation because of φ_*i*_,_delay_ (eq 2), increasing from **Zone 1** to **Zone 5**. For instance, φ_5_,_delay_ is 225 min longer than φ_1_,_delay_. Since **Zone 5** was subjected to the slowest *J*_EGF_ and longest φ_*i*_,_delay_, these factors suggest that cell activation and deactivation (which lowers CNR) for **Zone 5** were the most asynchronous of the zones in the LDA. The outcome would be suppression of the overall average CNR_*i*_ at each time point. To reevaluate peak activation, instead of averaging relative to a global *t* = 0, we synchronized cell activity by making *t* = 0 the time point at which an individual cell reached a CNR equal to or greater than one (see Materials and Methods). Originally, we were averaging CNRs as a regular time average, where the mean CNR for all cells in a zone was averaged at every time point. We wanted to account for the real-time activation when averaging CNR by syncing the activation start time. After syncing the starting time point of activation for each individual cell in the LDA, we recalculated the average CNR_*i*_ for only activating cells within each zone. Magnitude of the synced peak activation was approximately the same across all zones (±3.5% RSD) (Figure 3D). Interestingly, even after synchronizing cell activation times, the rate of activation (increasing CNR) and deactivation (decreasing CNR) appear to decrease with decreasing *J*_EGF_. This can be observed by the more gradual upward and downward slopes of the CNR around each zones’ respective peak (Figure 3D). These observations suggested that *J*_EGF_ had a direct impact on the activation and deactivation patterns of cells. Indeed, we found that there was a significant relationship between flux and activation and deactivation rates across all experiments (Figure S4).

### Threshold Concentration of Extracellular EGF Required for Activation of Intracellular Akt Depends on Cell Density

Next, we sought to identify a threshold concentration of EGF (EC50_EGF_) that prompts Akt activation in KTR cells in the LDA. We performed a dose-response experiment within the microchannel by increasing EGF concentration in a stepwise manner (Figure 4A) and compared low (<250 cells/mm^2^), medium (250–500 cells/mm^2^), and high (>500 cells/mm^2^) cell density groups (Figure 4B). The half maximal effective concentration for EGF (EC50_EGF_) was determined to be the point at which greater than 50% of the experimental sample population of cells had activated Akt (i.e., CNR > 1). Interestingly, we observed a positive correlation between cell surface density (σ) and EC50_EGF_ in the LDA (Figure 4C). We extracted EC50_EGF_ values for the varying cell densities and performed linear regression to determine a general equation for the EC50_EGF_ at any cell density (Figure 4C, eq 5). Notably, there would likely be limits with the graph approaching a small, positive value (lower limit) for EC50_EGF_ in low cell densities, and similarly, some large, finite value (upper limit) for EC50_EGF_ in increasingly high cell densities. The range of cell densities in the experiments presented appears to fall within the linear domain (Figure 4C, **solid red line**).

**Figure 4:**
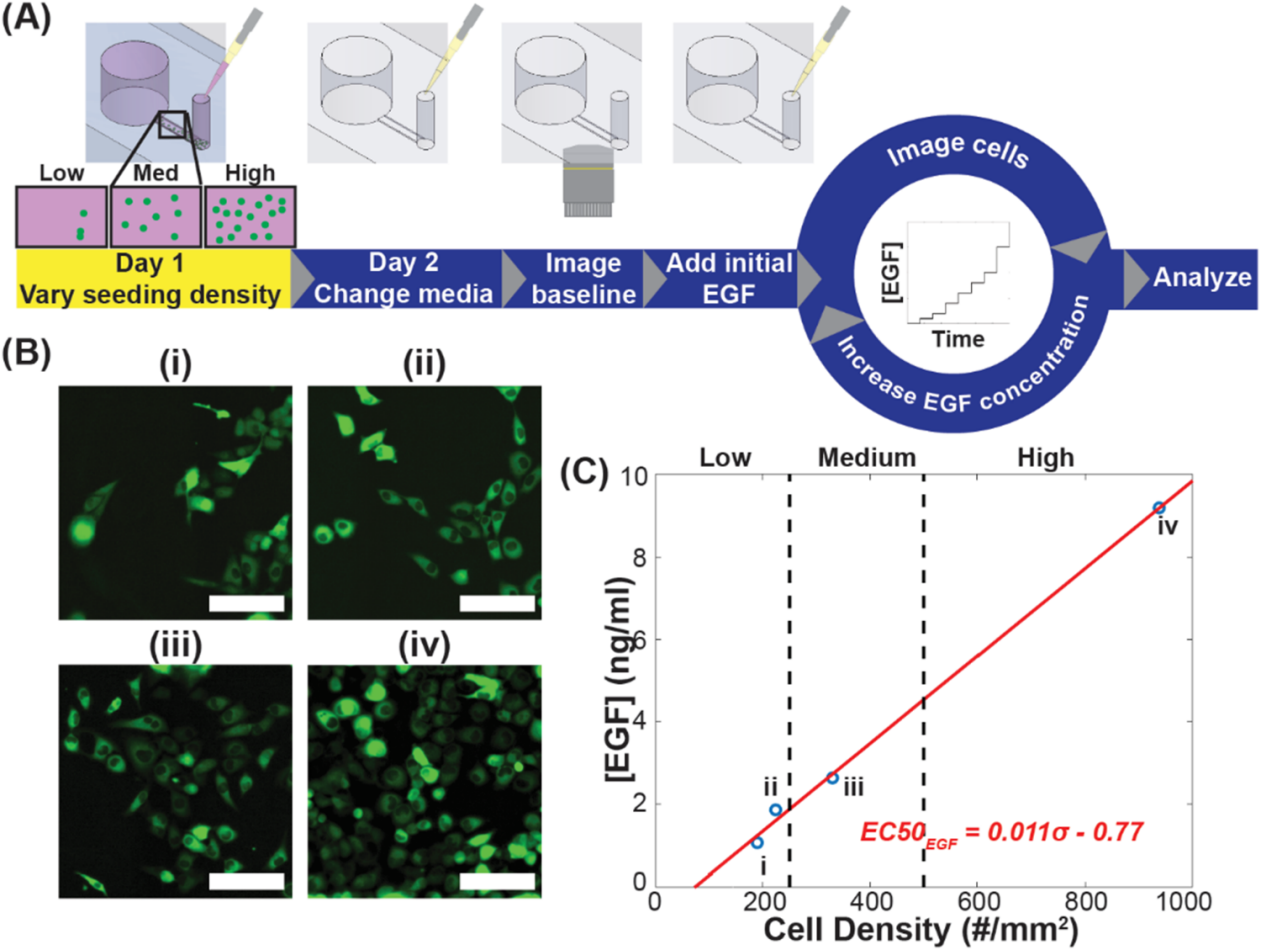
Determining half maximal effective concentration of EGF (EC50_EGF_) for Akt activation as a function of cell density. (A) The workflow for determining the EC50_EGF_ in the LDA. (B) Representative images of various cell densities. (i) and (ii), low cell densities (<250 cells/mm^2^) at 2 ng/mL of EGF. (iii) Medium cell density (between 250 and 500 cells/mm^2^) at 4 ng/mL of EGF. (iv) High cell density (>500 cells/mm^2^) at 10 ng/mL EGF (scale bar = 125 μm). (C) EC50_EGF_ as a function of cell density. The dashed vertical lines represent the divisions between low, medium, and high cell density ranges. The solid red line represents a linear fit; dashed red lines fall outside of the experimentally observed range. EC50_EGF_ is the effective EGF concentration for 50% Akt activation, σ is the cell density.

We benchmarked the EC50_EGF_ results in the LDA with studies in macroscale multiwell (24-well) plates and serial dilutions of EGF (Figure S5). Similar to experiments in the LDA (Figure 5), the EC50_EGF_ in 24-well plates was directly proportional to cell density. However, the EC50_EGF_ observed in the 24-well plates was ∼8× more sensitive to changes in cell density than those estimated within the LDA (Figure S5). For example, the EC50_EGF_ for an average cell density of 250 cells/mm^2^ was predicted to be 1.98 and 11.67 ng/mL in the LDA and 24-well plate, respectively. Moreover, the extracted values likely underpredicted the true EC50_EGF_ values within the 24-well plates. In 24-well plates, a bolus dose of 100 μL of 10× EGF was added to 900 μL of imaging media. Cells would initially be exposed to a concentration of EGF somewhere between the intended 1× and bolus 10× concentration due to unpredictable mixing. Thus, the LDA offered more predictable EC50_EGF_ values that were less sensitive to cell density.

**Figure 5:**
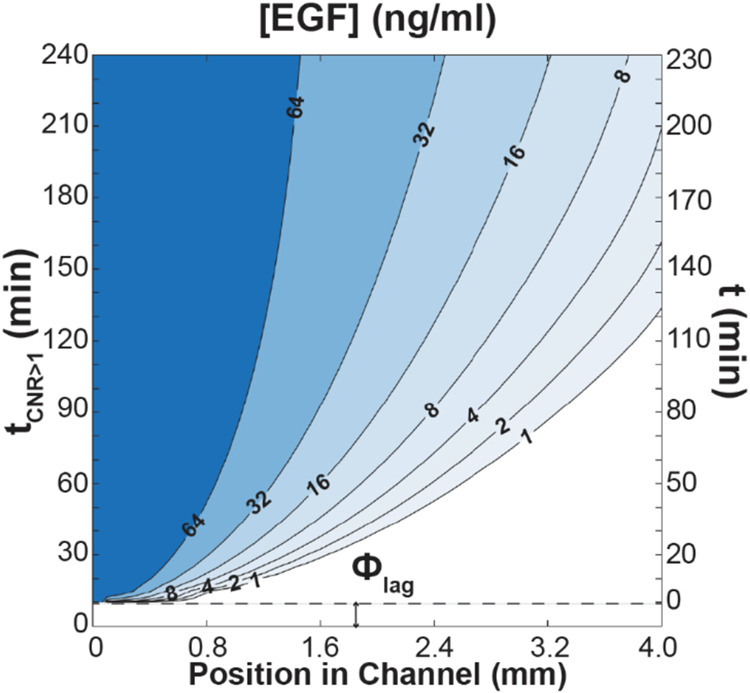
Contour map of EGF concentration as a function of time and channel position. The isolines represent theoretical EC50_EGF_ values in ng/mL. Akt KTRs had a delay upon exposure to EGF and observed KTR activation represented here by Φ_lag_.

### Building a Spatiotemporal Model to Correlate EGF Diffusion to Akt KTR activation

Once determining the values for EC50_EGF_ empirically (previous section), we constructed a spatiotemporal model to relate intracellular Akt KTR activation of individual cells with diffusion-limited EGF gradients in the LDA. This diffusion model was assumed to be one-dimensional and governed by Fick’s second law (eq 6). The diffusion coefficient was determined by the Stokes–Einstein equation (eq 4). As described earlier, the nonhomogeneous boundary condition due to a nonsteady source concentration promoted a numerical solution for Fick’s second law using the forward Euler method (eq 7). Using the approximation, we found *t*_EC50_ (Figure 5) which is the point where the concentration of EGF is equal to EC50_EGF_ and therefore has reached the threshold to activate Akt in the median cell. Furthermore, we observed an approximately 10 min delay from when cells are exposed to a stimulating level of EGF to activation of the KTR signal (CNR > 1). This 10 min delay was observed in 24-well plates when adding a 100 μL bolus of EGF to each well (Figure S5B). Due to the rapid introduction of EGF in the multiwell plates, we conclude that this time delay is the minimum time required between stimulation and activation. We denote this delay as the fixed parameter Φ_lag_. This parameter is an inherent property of the signal transduction dynamics of KTR cells and represents the time required from EGF-EGFR binding to translocation of the Akt KTR construct out of the nucleus. We applied Φ_lag_ to predict the median activation time:

3 $$t_{\mathrm{CNR}>1}=t_{\mathrm{EC}50}+\Phi_{\mathrm{lag}}$$

where *t*_CNR>1_ is the time at which the CNR is greater than one and the KTR is therefore considered activated. Using eq 6 for which eq 3 provides our time parameter, we created a contour map of EGF concentration as a function of time and lateral channel position (Figure 5). The isolines represent theoretical EC50_EGF_ values. For any given experiment, there should be a corresponding isoline that fits the activation pattern of the KTR across space and time if the assumption of pure diffusion holds true.

### Varying Source Concentration of EGF Alters Akt Signaling Activation and Deactivation

Once we established our model, we were equipped to predict the time of KTR activation for the median cell in the LDA based on the location of the cell and the EC50_EGF_. To challenge the robustness of our model, we assessed the impact of different rates of EGF concentration change on Akt activation and deactivation by introducing EGF at three different source concentrations: (1) 10, (2) 100, and (3) 1000 ng/mL (Figures 3C and 6A). For the experimental procedure, we followed the same protocol described in Figure 1B with the alteration of varying the concentration of EGF added to the inlet of the LDA (Figure 6B). Observed cell densities were 409, 367, and 846 cells/mm^2^ which yielded EC50_EGF_ values of 4.96, 3.27, and 8.53 ng/mL (eq 5) for the 10, 100, and 1000 ng/mL source concentration experiments, respectively. The isoline of each EC50_EGF_ was overlaid onto the respective heatmap (Figure 6C, **black dotted line**) as previously described (Figure 5). For the 10 ng/mL condition, the model only predicted activation up to Zone 3 within the 4 h experiment window, as seen by the isoline crossing the 240 min line at only the 2 mm position. This was observed in the heatmap (Figure 6C­(i)) as well as with the percentage of responding cells dropping below 20% by **Zone 3**. In comparison, cells are activated above 60% through **Zone 4** for the 100 ng/mL condition and above 60% for all zones in the 1000 ng/mL condition (Figure S6A). There was also spurious cell activation, which was observable in **Zones 3–5**, often before EGF was predicted to arrive. This outcome could be due to autocrine or paracrine signaling, which was seen to be proportional to the experimental cell density.^34–36^ Overall, the Akt activation profiles determined from our model qualitatively show good agreement with the experimental values, thereby indicating robustness of the prediction.

**Figure 6:**
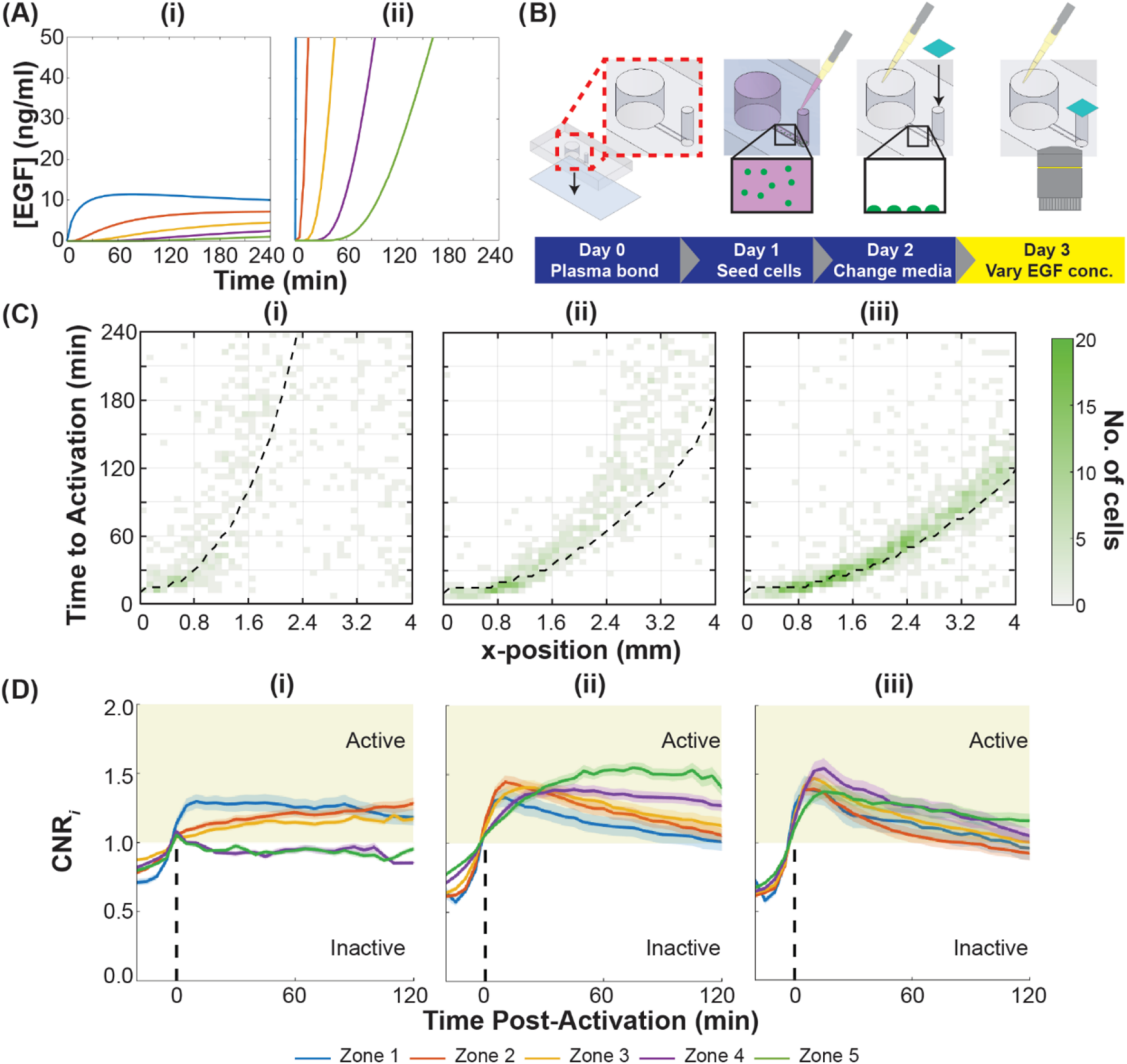
Impact of varying EGF source concentration on Akt activation. (A) EGF concentration changes over time for each zone with an EGF source concentration of (i) 10 ng/mL and (ii) 1000 ng/mL. (B) The experimental setup varies from Figure 1B to supply different source concentrations of EGF. (C) Isolines for EC50_EGF_ are plotted over heatmaps for Akt activation in individual cells from varying EGF source concentrations of (i) 10 ng/mL, (ii) 100 ng/mL, and (iii) A source of 1000 ng/mL. (D) Activation start times for individual cells are synced, and CNR_i_ is plotted over time for EGF source concentrations of (i) 10 ng/mL, (ii) 100 ng/mL, and (iii) 1000 ng/mL.

As previously stated, we observed an impact on Akt activation and deactivation due to varying *J*_EGF_ (EGF flux). Higher source concentration resulted in larger *J*_EGF_ across all zones (Figures 3C and 6B). *J*_EGF_ also varied zonally in the LDA. Smaller *J*_EGF_ values produced more sustained activation and slower deactivation of Akt in subpopulations of cells in **Zones 1–3** of the 10 ng/mL condition and **Zones 4–5** of the 100 ng/mL condition, as seen by the flatter slopes after activation (Figure 6D).

### High Cell Density Increases Akt Activation Starting Time and Magnitude

Next, to investigate the impact of cell density on Akt signaling dynamics, we seeded the LDA with either low (<250 cells/mm^2^), medium (more than 250 or less than 500 cells/mm^2^), or high (>500 cells/mm^2^) cell concentrations (Figure 7A,B). Since cell adherence to the device surface was variable, devices were sorted postimaging based on actual coverage. In each case, the source concentration of EGF was the same at 100 ng/mL. Average densities were 122, 353, and 547 cells/mm^2^ for the low-, medium-, and high-density cases, respectively. Relative standard deviations of the density across zones were 12.6, 15.7, and 6.2% for each case, respectively (Figure S7D–F). We used eq 5 to calculate a predicted EC50_EGF_ of 0.57, 3.11, and 5.25 ng/mL for the low, medium, and high densities, respectively. We again plotted the EC50_EGF_ isolines over the respective heatmaps of individual cell Akt activation (Figure 7C). The isolines showed good agreement with the KTR activity, supporting our observation that higher cell densities result in higher EC50_EGF_ values (Figure 7C). This outcome is evident by the increased time to activation values for the highest cell density condition, despite having the same EGF source concentration as the lower conditions.

**Figure 7:**
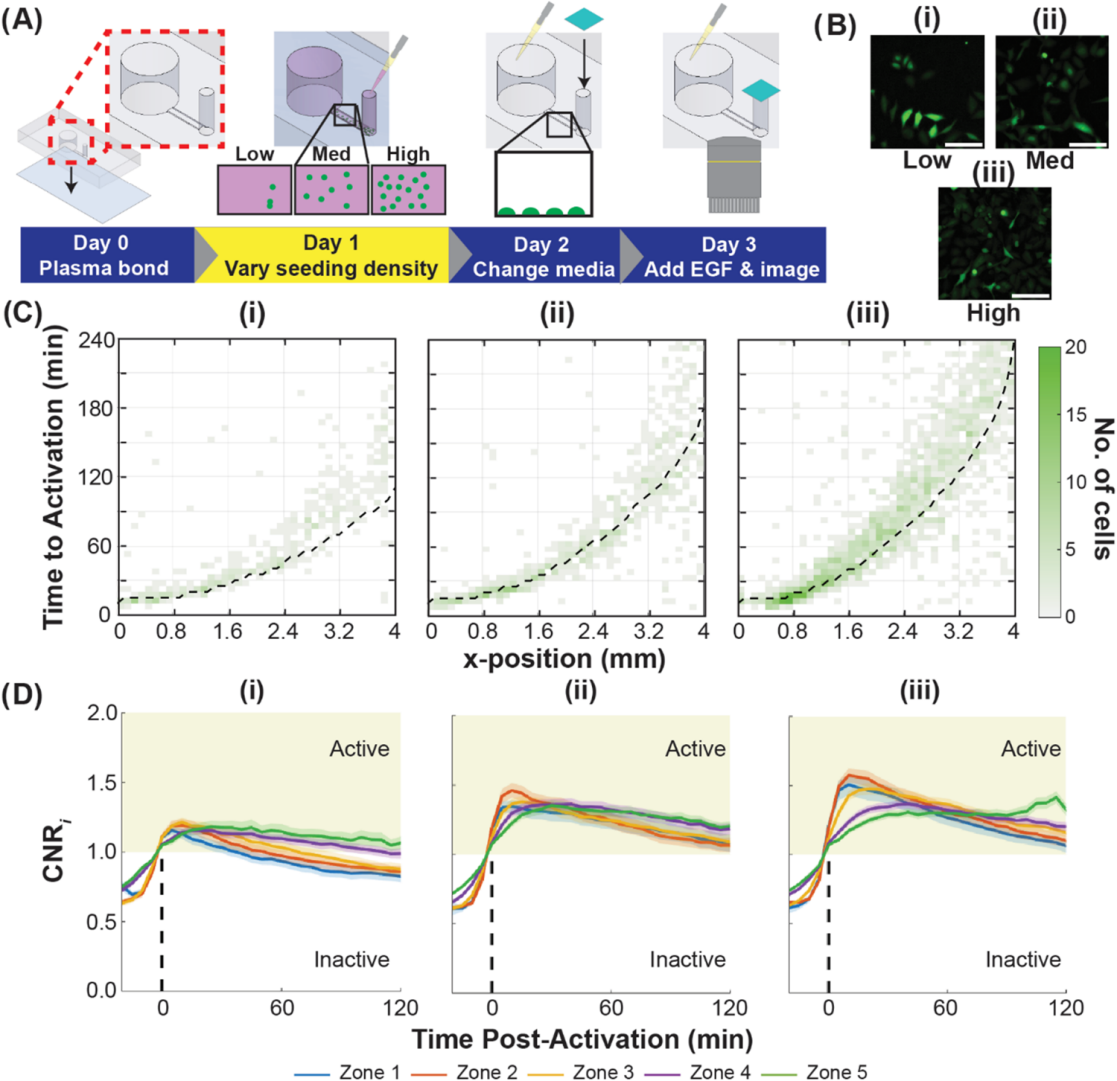
Akt activation patterns change with increasing cell density within the LDA. (A) The procedure for the LDA is altered from Figure 1B to vary the cell density in the microfluidic channel by changing the number of cells seeded. (B) Representative images of various cell densities. (i), Low cell densities (<250 cells/mm^2^). (ii), Medium cell density (between 250–500 cells/mm^2^). (iii), High cell density (>500 cells/mm^2^). (C) Isolines for EC50_EGF_ are plotted over heatmaps for Akt activation in individual cells for (i) low, (ii) medium, and (iii) high cell densities within the LDA. (D) Activation start times for individual cells are synced and CNR_i_ is plotted over time for (i) low, (ii) medium, and (iii) high cell densities.

We determined that activation and deactivation patterns corresponded to *J*_EGF_ values for different cell densities in the LDA. Similar to previous observations (Figure 3D), the lower *J*_EGF_ values in the LDA correlated to more sustained activation times shown by smaller downward slopes after activation (Figure 7D, **Zones 4** and **5**). Alternatively, higher *J*_EGF_ values correlated with more rapid deactivation with larger downward slopes after initial activation (Figure 7D, **Zones 1–3**). Additionally, higher cell densities resulted in higher peak activation in **Zones 1–3** (Figure 7D). The relationship between cell density and peak activation was found to be significant, while flux had no significant correlation with peak magnitude (Figure S4). We anticipate that this is due to increased autocrine and paracrine signaling from the increasing cell density. For instance, Figure 7D­(iii) represents the highest cell density and displays the largest magnitude and most sustained Akt signaling. Even **Zones 4–5**, where EGF concentrations will be lowest, in the medium- and high-density cases, show larger CNRs than any zones in the low-density case (Figure 7D). This response suggests that EGFR-independent pathways are activating Akt. These results demonstrate that both cell density and spatial position within the channel profoundly influence Akt signaling dynamics.

### Inhibiting Endocytosis Does Not Alter EGF Diffusion but Modulates Akt Signaling Duration

Because of the dependence of Akt activation on cell density, we sought to determine if endocytosis of the EGF receptor (EGFR) could play a role in cell density-dependent behavior. To evaluate whether clathrin-mediated endocytosis (CME) influences EC50_EGF_ and hence Akt activation, we pretreated KTR cells with cell membrane-permeable Dyngo-4a to block vesicle scission.^37,38^ Dyngo-4a diffused through the microfluidic channel 1 h prior to adding 100 ng/mL of EGF and subsequent imaging (Figure 8A). Dyngo-4a is a small-molecule inhibitor of dynamin, which is the GTPase that binds to the neck of a vesicle and aids in pinching off the invaginated vesicle during CME (Figure S8). Therefore, since EGF-EGFR complexes are not internalized or recycled to the cell surface, EGF consumption should be reduced. We hypothesized that this would negate the relationship between cell density and EC50_EGF_ because less receptor recycling would reduce EGF consumption. Importantly, Dyngo-4a does not impair the activation of Akt as it has been shown that this pathway is clathrin-dependent, but not dynamin-dependent.^39^ The cell densities for each case were 167, 250, and 579 cells/mm^2^ with EC50_EGF_ values of 1.07, 1.98, and 5.60 ng/mL, respectively. Ultimately, there was no effect on EC50_EGF_ across the varying cell densities (Figure 8B). eq 5 was still sufficient for calculating EC50_EGF_. Interestingly, the percentage of activated cells remained high in **Zone 1** but decreased in a near-linear fashion (Figure S6C and Table S3). The Dyngo-treated cells also appeared to have steeper deactivation compared with non-Dyngo-treated cells (Figure 8C). The reduction in activation duration corroborates reports that CME is essential for sustained EGFR signaling.^40^ Cell density is the most influential factor for the variation in the EGF activation threshold for predicting Akt activation in the LDA. Overall, the results suggest that endocytosis contributes to sustained signaling rather than signal initiation.

**Figure 8:**
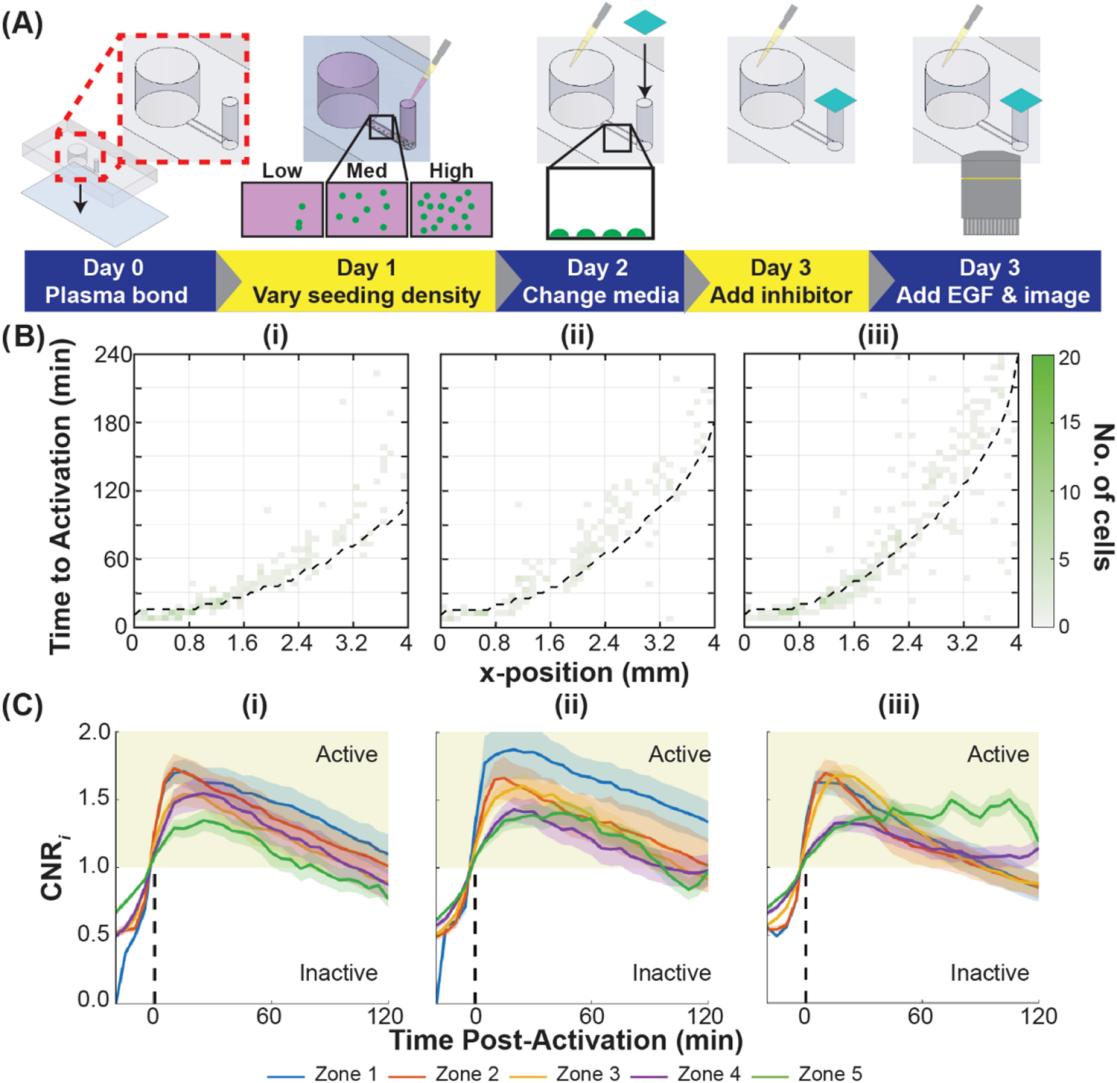
Inhibiting endocytosis has no apparent effect on Akt activation. (A) An extra step is added to the LDA 1 h prior to imaging to introduce an endocytosis inhibitor to the microchannel. (B) Cell density is varied, and Dyngo-4a is introduced to the microchannel 1 h prior to adding 100 ng/mL of EGF and imaging. There appears to be no effect from inhibiting clathrin-mediated endocytosis on Akt activation in individual cells in **(i)** low, **(ii)** medium, nor **(iii)** high cell densities. (C) Activation start times for individual cells are synced, and CNR_i_ is plotted over time for **(i)** low, **(ii)** medium, and **(iii)** high cell densities.

## Discussion

Chemical gradients play a key role in many cellular processes and functions, such as cell signaling and chemotaxis (i.e., cell migration in response to an established gradient), *in vivo*.^41^ Microfluidic platforms have emerged as important tools for studying gradient-dependent phenomena *in vitro*.^42^ Here, we describe a lateral diffusion assay (LDA) as a new microfluidic-based and pump-independent platform to investigate how diffusion-based gradients of epidermal growth factor (EGF) regulate spatiotemporal Akt signaling in triple-negative breast cancer (TNBC) cells. The optical transparency of the LDA in combination with kinase translocation reporters (KTRs) enabled real-time visualization and quantification of Akt activity under well-controlled molecular gradients. This approach addresses a critical gap in conventional assays, which lack spatial resolution and rely on bolus ligand delivery that obscures the gradient-dependent signaling dynamics.

Parallel flow (e.g., multiple laminar streams) and upstream mixer (i.e., Christmas tree structure) are well-established microfluidic methods for generating stable and precise biomolecular gradients.^43–46^ These configurations typically have source and sink channels under continuous flow from external pumps. The nature of the gradient can be controlled and reconfigured by adjusting input flow rates and redesigning the upstream mixing channels.^47^ In contrast, the simplicity and low maintenance operation of the LDA is an advantage in comparison to most microfluidic gradient models, especially pump-based ones that are prone to operational failures due to air bubble formation in the tubing interface between pumps and microchannels. However, unlike pump-based systems, the LDA is incapable of reconfigurable gradients through active switching and reversal methods. Indeed, our group has recently used pump-based microfluidic gradient systems for studying the effects of abrupt changes in local ion concentrations on molecular sensors^48^ and reversal of the orientation of a chemotactic gradient on the polarity of migrating cells.^46^ Another consideration for the convection-neutral nature of the LDA is potential conditioning of cells by autocrine and paracrine factors. To this end, we have previously shown that microfluidic gradients can be established *in vitro* and independent of pumps through precise positioning of chemokine CXCL12 source and sink (or scavenging) cells that recapitulate the cancer-stroma microenvironment.^49^ Future studies could integrate biomolecular source and sink cells, which could further enhance the physiological relevance of the LDA platform. Finally, unlike pump-based microfluidics, the passive (or “set it and forget it”) operation of the LDA is conducive to automation with automated liquid handlers that are mainstays of contemporary and industrial pharmaceutical drug discovery workflows.^50^

Introduction of solutes to most microfluidic-based systems requires the addition of some finite droplet of liquid to the inlet port. Due to surface tension, any droplet or dome of liquid on the port imparts pressure as governed by the Young–Laplace equation, with smaller droplets containing higher pressures.^51^ The pressure gradient introduced from a liquid droplet would therefore result in a convective flow in devices with other open ports. Shear stress imparted by convective flow has been shown to influence intracellular kinase signaling, thus confounding effects due to biomolecular gradients alone.^18,19^ Previous designs have used methods such as upstream networks of microchannels or porous membranes to increase resistance to the propagated convective flow.^44,52^ The LDA circumvented this challenge by simply occluding the outlet port and relying on the incompressible nature of fluids to negate the droplet from imparting a convective flow in our channel. The agreement of experimental and numerical methods confirmed that convection was negligible, and the LDA produced diffusion-driven gradients.

Leveraging diffusion-limited transport in the LDA, we assessed several spatially regulated determinants of Akt signaling: (i) cell density, (ii) distance from the EGF source, and (iii) flux of the EGF. We observed a positive correlation between the density of cells and the EGF threshold concentration for Akt activation in the median cell population (EC50_EGF_, Figure 4C). Notably, EC50_EGF_ was eight times more sensitive in 24-well plates versus the LDA (Figures 4 and S5). We believe that the lower EC50_EGF_ values within the LDA could be due to one of two factors: surface area to volume ratio (SA/V) or material differences in cell culture systems. The LDA has an SA/V of 1.01 compared to 0.31 for a well in the 24-well plate. The higher SA/V leads to higher uptake of nutrients, increased paracrine and autocrine signaling, and decreased clearance of waste products.^53^ Hence, the LDA would have a higher nutrient uptake and more even distribution of EGF, leading to the lower observed EC50_EGF_ values when compared to those of the 24-well plate. Alternatively, polydimethylsiloxane (PDMS) used to fabricate the LDA has drawbacks, such as residual, uncured oligomers that may interfere with signaling or optically transparency compared to polystyrene (PS) used in 24-well plates.^53,54^

A previous study that also used PDMS-based microdevices to study KTR ERK signaling in mammary epithelial cells concluded that higher rates of EGF introduction resulted in a higher percent population response of ERK as well as longer signaling duration.^30^ In accordance with these results, we saw more uniform signaling in Akt KTR cells for both higher EGF fluxes and higher source concentrations of EGF. Unique to our configuration, we were able to directly compare the effects of different EGF fluxes in a single experiment (Figures 3C and 6A). We observed that a lower EGF flux correlated with slower activation and deactivation times (Figure S4). Slower deactivation is likely due to less desensitization to EGF from reduced EGFR internalization in the presence of a lower EGF concentration.

While cell density had a significant impact on the EGF activation threshold, the impact of cell density on spatial Akt signaling dynamics in the diffusion-limited setting of LDA was unclear. The diffusion-limited experiments were consistent with the microchannel threshold experiments, as the empirically derived relationship between cell density and EC50_EGF_ (eq 5) was able to predict accurately the dependence of EC50_EGF_ on cell density. There were clear differences in the time to activate and the magnitude of the CNRs. Higher cell densities took longer to activate, had a higher CNR, and displayed more sustained Akt activation. Higher CNRs and more sustained activation in more densely packed channels indicate that paracrine and autocrine signaling result in more off-target Akt activation.^34–36^ Moreover, many factors released by MDA-MB-231 cells contribute to Akt signaling independently of EGF.^55–59^ Paracrine and autocrine signaling can also explain the increase in the EGF threshold at higher cell densities. With more non-EGF paracrine and autocrine factors released in the supernatant liquid, the cells display a decreased sensitivity to increasing EGF concentrations.

We hypothesized that the longer time to activation could be due to the higher consumption of EGF by the higher density of cells. MDA-MB-231s have a high expression of EGFR with a fast recycling rate,^32^ so sequestering of EGF-EGFR complexes through internalization could result in limited EGF availability for highly dense cell groups. To minimize effects on cell signaling, we avoided direct inhibition of clathrin, as it has been implicated as a requirement for EGF-Akt phosphorylation.^39^ Instead, we utilized a dynamin inhibitor, Dyngo-4a, which ultimately had no effect on the threshold concentration, suggesting another mechanism for the cell density dependence. Unexpectedly, applying Dyngo-4a to the KTR cells caused the average CNR to increase in **Zone 1** and decrease in a near-linear fashion in subsequent zones. CNRs were approaching 1.8 compared with 1.5 seen in other results. The increase in Akt activation is likely from persistent activation caused by the dynamin inhibition.^60^ This CNR value matched more closely to what we observed in 24-well plate experiments.

While the simplicity and robustness of the LDA enable valuable insight into the spatiotemporal signaling dynamics of Akt in TNBC in response to controlled delivery of biomolecular gradients, a limitation is that it lacks several factors of the tumor microenvironment (TME) that affect gradients and kinase signaling. Blood and lymphatic vessels control interstitial flow and diffusion in the TME in a physiological setting.^61^ Vascular endothelial cells also secrete cancer migration-inducing paracrine factors whose abundance is shaped by mass transport.^62^ Furthermore, structural extracellular matrix (ECM) proteins and GAGs, such as collagen and hyaluronic acid, influence mass transport properties and influence cell signaling.^63,64^ Moreover, our studies assessed individual cancer cells while these cells assembled in 3-D as either spheroids or organoids are known to be better predictors of drug response.^65^ Nevertheless, the LDA is sufficiently modular such that it can incorporate other key elements of the TME and their physical environment, thereby opening new potential insights in future studies.

## Conclusions

We demonstrated the utility of the LDA to repeatedly produce gradients from a singular droplet of fluid containing small molecules. The process is simple and can be easily combined with fluorescent reporters in live cells to obtain dynamic readouts of cell signaling. Furthermore, the gradients allowed us to observe patterns in Akt signaling that correlated with (i) cell density, (ii) distance from the EGF source, and (iii) flux of EGF. We observed that inhibiting EGFR internalization did not affect the diffusion dynamics of EGF. Ultimately, spatial information in tumors is vital to understanding environmental cues, drug/cytokine gradients, and cell–cell communications; hence, future studies and assays should acknowledge the importance of these factors to understanding cell signaling responses.

## Materials and Methods

### Triple-Negative Breast Cancer Cell Culture and Kinase Translocation Reporter

The triple-negative breast cancer cell line MDA-MB-231 stably expresses a kinase translocation reporter (KTR) for Akt. Briefly, the KTR contains a substrate for the Akt. Phosphorylation of a nuclear export signal by binding of Akt to the substrate allows the KTR to be transported to the cytoplasm to indicate an active state. Conversely, dephosphorylation of a nuclear localization signal transports the KTR to the nucleus when it is inactive. An enhanced green fluorescent protein (EGFP) is attached to the KTR for real-time visualization via fluorescent microscopy.^13,17^ A red fluorescent protein (mCherry) is attached to histone H2B to locate the nucleus and measure the cytoplasmic-to-nuclear ratio (CNR) of the KTR.

The MDA-MB-231 cells were maintained in Dulbecco’s Modified Eagle Medium, high glucose (DMEM, Gibco, Waltham, MA) supplemented with 10% fetal bovine serum (FBS, Avantor, Radnor, PA) and 1% penicillin-streptomycin-glutamine 100× (Gibco, Waltham, MA). Cells were cultured in a humidified incubator at 37 °C and 5% CO_2_. Cells were harvested and passaged every 2–3 days. The absolute passage number was not tracked; however, all experiments were performed using cells less than 3 months (∼30 passages) from a frozen vial. Culture media was aspirated from the flask and cells were washed with 1× Dulbecco’s Phosphate-Buffered Saline (DPBS) without Mg/Ca (Gibco, Waltham, MA). Next, cells were detached from the culture flask with 0.05% trypsin EDTA 1× (Gibco, Waltham, MA), and trypsin was neutralized with the culture media. Cells were centrifuged at 200 rcf for 5 min at room temperature. The media-trypsin mixture was aspirated, and the cell pellet was resuspended in culture media. Approximately 1 × 10^6^ cells were replated on a T-75 culture flask.

### Microfluidic Device Preparation

The straight-channel microfluidic devices were made using a silicon wafer master mold. Photolithography was used to create a master mold with channel dimensions of approximately 150 μm × 4 mm × 500 μm (height × length × width). Soft lithography was then used to create the final PDMS devices. Briefly, PDMS base and elastomer curing agent were mixed at a 10:1 ratio, respectively. The PDMS mixture was then degassed to remove air bubbles and poured over the master mold. The PDMS was then kept in a 65 °C oven on the master mold overnight. Once the PDMS was a solid elastomer, it was removed from the wafer. Individual devices were cut out from the template. Each device was bound to a glass slide using a plasma oxidizer (Harrick Plasma, Ithaca, NY, Figure 1B, **Day 0**).^66^

### Experimental Confirmation of Diffusion in the Lateral Diffusion Assay

We used 10 kDa FITC-dextran (Sigma-Aldrich, St. Louis, MO) to experimentally confirm the diffusion-limited transport within the LDA. Images were acquired using a Nikon Eclipse Ti2 microscope with a point scanning confocal (Nikon AXR). A 4× objective was used to capture a single field of view from the inlet to the outlet port. A 488 nm excitation laser was used with 10% power and 60× gain.

To measure diffusion, the device was filled with 1× DPBS, the outlet was covered with a piece of microfluidic tape (3M, St. Paul, MN), and a 10 μL droplet of 10 mg/mL of 10 kDa FITC-dextran mixed in 1× DPBS was added to the inlet to dilute it to 1 mg/mL. The device was then imaged every 5 min for 4 h. The experimentally generated concentration curves were compared with theoretical concentration curves using the theoretical diffusion coefficient of 10 kDa FITC-dextran. Theoretical diffusion coefficients (*D*) are calculated using the Stokes–Einstein equation:

4 $$D=\frac{k_{B}T}{6\pi \mu R_{h}}$$

where *k*_B_ is the Boltzmann constant, *T* is temperature, μ is solvent viscosity, and *R*_h_ is the hydrodynamic radius of the solute. The hydrodynamic radius of the 10 kDa FITC-dextran was specified as 2.3 nm by the manufacturer, resulting in a diffusion coefficient of 9.37 × 10^–11^ m^2^/s at 22 °C and assuming a viscosity of 1 cP. For modeling EGF diffusion, a hydrodynamic radius of 1.4 nm was assumed. This resulted in a diffusion coefficient of 1.62 × 10^–10^ m^2^/s at 37 °C and the viscosity of 1 cP.

### Seeding Cells in the Lateral Diffusion Assay

MDA-MB-231 KTR cells were harvested from the culture flask and suspended in cell culture media at approximately 750 cells/μL. At the 750 cells/μL density, we would observe 60–70% confluence in the microchannel of the LDA. For cell density experiments, cells were suspended between 500–2000 cells/μL where 500 cells/μL showed ∼20% confluency and 2000 cells/μL showed greater than 80% confluency after 24 h. Device surfaces were treated with fibronectin (Sigma-Aldrich, St. Louis, MO) at 100 μg/mL for 25 min to enhance cell attachment.^67^ A cell suspension of 20 μL was pipetted through the microfluidic channel using the small outlet port. Excess liquid was removed by pipette at the inlet and outlet to confine cells to the channel and prevent the buildup of cells in the port. After the excess liquid was removed, cells were allowed to attach for 4 h in a humidified incubator at 37 °C and 5% CO_2_. After 4 h, 100 μL of culture media was added to the large inlet port (Figure 1B, **Day 1**). After 24 h, the culture media were aspirated and replaced with Fluorobrite DMEM (Gibco, Waltham, MA) supplemented with 0.1% FBS and 1% Pen-Strep-Glutamine (imaging media). This was meant to condition cells and prevent high levels of FBS from activating the KTR as well as omitting phenol red from the media to improve background interference when imaging. Additionally, a small piece of microfluidic tape (3M, Saint Paul, MN) was placed over the outlet port of the channel (Figure 1B, **Day 2**). The inlet of the device was emptied and then replenished with 90 μL of imaging media, and the devices were then placed back into the incubator overnight before imaging.

On the day of imaging, devices were taped down to the bottom of a 100 mm culture dish. The dish was lined with wet Kimwipes to keep devices hydrated for the duration of the experiment. After the incubator on the microscope reached 37 °C and 5% CO_2_, the dish was placed on the stage. EGF (PeproTech, Waltham, MA) was prepared in the imaging media. EGF working concentrations were prepared at 0.1, 1, and 10 μg/mL for low, medium, and high concentration experiments, respectively. Once the working concentration of EGF was prepared, 10 μL of the solution was added to the inlet of the device. The inlet was prefilled with 90 μL of imaging media, resulting in a further 10× dilution of the EGF working solution and final source concentrations for low, medium, and high concentrations of 10, 100, and 1000 ng/mL, respectively. Imaging began immediately after adding the EGF (Figure 1B, **Day 3**).

For inhibitory experiments using Erlotinib (Cayman Chemical, Ann Arbor, MI), a working concentration of 100 μM of Erlotinib was prepared by diluting a stock concentration of Erlotinib in Fluorobrite DMEM. The Erlotinib was delivered simultaneously with 1000 ng/mL of EGF prior to imaging, and the concentrations were diluted 10× to 10 μM and 100 ng/mL, respectively.

### Dilutions of EGF from Stock Concentrations

EGF (PeproTech, Waltham, MA) was reconstituted in sterile 1× DPBS and stored in aliquots at a stock concentration of 100 μg/mL. For all LDA experiments, a fresh aliquot of stock solution was diluted to a working concentration that was 10× of the final concentration. For the 10 ng/mL source condition, 1 μL of stock was diluted in 999 μL of imaging media to achieve a 100 ng/mL working solution from which 10 μL was added to the 90 μL of media in the inlet port, resulting in a final concentration of 10 ng/mL. For the 100 ng/mL source condition, 10 μL of stock was diluted in 990 μL of imaging media for a 1000 ng/mL working solution. For the 1000 ng/mL source condition, 10 μL of stock was diluted in 90 μL of imaging media for a 10,000 ng/mL working solution.

### Imaging MDA-MB-231 KTR Cells in Lateral Diffusion Assay

A fully automated Lionheart FX microscope (BioTek, Winooski, VT) with Gen5 software was used to capture all fluorescent and timelapse images of MDA-MB-231 KTR cells. A linear array of six images was taken on a 10x objective. This array captures the entire length of the microfluidic channel (Figure S7). Images were captured on two channels: the green channel captures the EGFP (Ex/Em: 469/525) of the KTR, and the red channel captures the mCherry of the nucleus (Ex/Em: 531/593). The microscope stage was equipped with a stage incubator to maintain the temperature at 37 °C and 5% CO_2_. Images were taken every 5 min on both channels and saved directly as tiff files for later analysis.

### Extracting Cytoplasm-to-Nuclear Ratios

Cells were identified and segmented using Cellpose and a custom human-in-the-loop trained model.^68,69^ To generate the custom model, five images were taken of the KTR cells using the same microscope (Lionheart FX) and used as training images. The base “cyto” or “nuclei” models that is shipped with Cellpose were used as starting points. The images were iteratively segmented and corrected by the user until all of the cells in every image were accurately identified. This resulted in two custom models: one to segment the nucleus and one to segment the cytoplasm. The segmented masks were then imported into bTrack for individual cell tracking.^70^ The tracked cell masks were then used to calculate the CNR of reporter fluorescence. Briefly, the nuclear masks were used to extract the average nuclear fluorescent intensity. To obtain the cytoplasmic intensity, the nuclear masks were dilated by 4 pixels (∼12 μm). The original nuclear mask was subtracted from the dilated mask, creating a “cytoplasmic ring.” The ring was then eroded by 1 pixel to ensure no overlap with the nucleus, with the final ring being ∼3 μm from the nuclei mask and ∼6 μm in thickness. The newly created cytoplasmic ring mask was used to obtain the average cytoplasmic fluorescent intensity. The fluorescent intensity from the cytoplasm was compared to that of the nucleus to get the CNR. A CNR above one meant that there was more KTR in the cytoplasm, indicating an active state.

### Determining Threshold Concentration of EGF for Akt Activation

Cells were seeded in devices as previously mentioned with varying densities. For the purposes here, we excluded the microfluidic tape from the devices so that we could rapidly flow EGF through our channel. Imaging was set up in the same way as the previous section on the Lionheart FX. A 5 μL drop of EGF at 20 ng/mL was added to the small port and passive pumping allowed it to rapidly flow through the channel. Images were taken manually 10 min later, as this is the approximate time dynamic of the EGF/EGFR pathway to activate Akt. This process was repeated for eight steps of EGF addition to achieve sequential device concentrations of 1, 2, 4, 6, 8, 10, 15, and finally 100 ng/mL. Concentration was then plotted against the percent activation of Akt for each cell density. An activation threshold was determined for the point at which over 50% of cells reached a CNR above 1.

Linear regression analysis (Figure 4C) yielded the following equation:

5 $${\mathrm{EC}50}_{\mathrm{EGF}}=0.011\sigma -0.77$$

where EC50_EGF_ is the EGF concentration (ng/mL) where 50% of cells activate (CNR goes above one), and σ is the average surface density of the cells (# of cells/mm^2^) in the LDA. Eq 7 was then used to calculate the time at which each x-position in the LDA reaches the EGF threshold concentration based on the EGF source concentration and plotted as a blacked dashed line over the Akt activation heatmaps.

### Spatial Mapping of KTR Cell Response

To produce the spatial and temporal mapping of the cell responses, both the location and activation time were extracted for each cell from the timelapse movies. Centroid locations of cells were extracted from the segmented masks (see Extracting Cytoplasm-to-Nuclear Ratios) and based on the first observed frame of each cell.

For average CNR plots, cells were grouped into distinct zones on the basis of their initial centroid location. The CNR was calculated for all cells within each zone and averaged at every time point (5 min). The average CNRs were then plotted over the duration of the experiment (4 h) for each respective zone.

For syncing activation start time in CNR plots, cells were again grouped into distinct zones based on the initial centroid location. Before plotting average CNR, we cycled through each cell in a zone to determine the time at which that cell first reached CNR > 1. This was then made the first time point for that cell. After syncing activation start time, CNRs were averaged at the new initial time and subsequent time points (every 5 min) and plotted for 2 h.

For activation heatmaps (or 2D histograms), the time to activation was calculated for each cell by finding the first instance of a CNR above 1. This time was paired with the cell’s centroid location and assigned into 2D bins of height and width of 5 min × 100 μm, respectively. The activation times for all KTR cells were binned and plotted on a two-dimensional histogram, or heatmap. The heatmaps intensity indicates the total number of observed cells belonging to each bin.

### Diffusion Model for Extracting Experimental Transport Properties

Diffusion in the LDA is assumed to be one-dimensional and governed by Fick’s second law:

6 $$\frac{\partial C}{\partial t}=D\frac{\partial^{2}C}{\partial x^{2}}$$

where $\frac{\partial C}{\partial t}$ is the change of the gradient over time, *D* is the diffusion coefficient (calculated by eq 4), and $\frac{\partial^{2}C}{\partial x^{2}}$ is the derivative of the spatial concentration gradient. The forward Euler method was used to calculate the transport of solutes within the microchannel device by solving Fick’s second law. The discretized form of the equation, assuming no convection, is given as

7 $$c_{t+1,x}=c_{t,x}+\frac{D\Delta t}{{\Delta x}^{2}}\left(c_{t,x+1}-{2c}_{t,x}+c_{t,x-1}\right)$$

where *c* is concentration, *t* is time, *x* is position in the channel, and *D* is the diffusion coefficient of the solute. For all models, we assumed a hydrodynamic radius of 1.4 nm for EGF, resulting in a diffusion coefficient of 1.6 × 10^–10^ m^2^/s at 37 °C and viscosity of 1 cP. A custom MATLAB script was used to calculate the theoretical concentration curve over the length of the channel (4 mm) and over the duration of an experiment (4 h). The time to reach a minimum concentration was generated by finding the first time step at which c was greater than the minimum concentration (*c*_min_) for each spatial x-location. *C*_min_ was determined by dosage experiments described in Determining Threshold Concentration of EGF for Akt Activation.

### Pretreatment of 231-KTRs with Endocytosis Inhibiting Agents

To study the impact of endocytosis on the diffusion of EGF, MDA-MB-231 KTR cells were pretreated with an endocytosis inhibitor, Dyngo-4a (Selleckchem, Houston, TX), which inhibits dynamin.^71^ Dyngo-4a stock was stored in aliquots of 10 μL at 5 mM in −20 °C. Dyngo-4a was prepared in working concentrations at 100 μM by adding 10 μL of stock to 490 μL of imaging media. It was diluted by 10× when added to the media of the inlet in the LDA. The choice of 10 μM final concentration was based on previous work, which showed inhibition of clathrin-mediated endocytosis at 5 μM concentration.^71^ Dyngo-4a was added to the LDA 60 min prior to EGF addition and imaging (Figure 5A, **Day 3**). Following the pretreatment, EGF was added to a final concentration of 100 ng/mL, and imaging commenced.

### Assessing Cell Viability Using Trypan Blue Stain

The KTR cells used in this study are genetically encoded with a green and red fluorophore for Akt and the nucleus, respectively. This limited our ability to use traditional live/dead staining kits that typically include a green and red signal. To circumvent this dilemma, we used Trypan blue to assess cell viability in the LDA. Cells were seeded in the device as described in Seeding cells in the lateral diffusion assay. After 24 h, the culture media were exchanged for imaging media. Following another 24 h, the Trypan blue stain was introduced. As a positive control, devices were heat-treated at 65 °C for 2 min to induce cell death. Test devices were maintained at 37 °C. A droplet of 5 μL of Trypan blue was added to the device. After 1 min, the excess was washed out of the channel using fresh imaging media. Devices were imaged with a 4× objective using an EVOS XL Core microscope.

### Statistical Analysis

Concentration curves of FITC-dextran are plotted as mean ± SD (shaded region) across *n* = 4 devices. Time-course plots of the cytoplasmic-to-nuclear ratio of cells are plotted as the mean with shaded 95% confidence intervals across cells. Independent experimental replicates refer to separate LDA devices prepared on separate experimental days, with n numbers listed in Table S3 for each condition. For correlation analysis, a nonparametric Spearman correlation was used with a threshold α = 0.05.

## Supplementary Material



## Notes

Diffusion source analysis, cell viability data, peak CNR analysis, flux-signaling correlation, comparative well plate data, activation percentages, representative images for data selection, diffusion coefficients, time delay data (PDF)
