# Supplementary material for: Quantitative Spatiotemporal Analysis of Intracellular Kinase Activity in Metastatic Breast Cancer Cells Using a Microfluidic-Based Lateral Diffusion Assay
Source: Anal Chem. 2026 Jun 21;98(25):18607–22. doi: 10.1021/acs.analchem.6c01060 (PMC13325445; doi:10.1021/acs.analchem.6c01060)
Supplement: Supplementary file 1 [file ac6c01060_si_001.pdf]

Supporting Information

Quantitative spatiotemporal analysis of intracellular kinase activity in metastatic breast cancer cells using a microfluidic-based lateral diffusion assay

Brendan T. Fuller[1], Travis H. Jones[2,*], Emily T. Chan[3,4], Malcolm W. D'Souza[5], Kathryn E. Luker[6,7], Gary D. Luker[6,8,], Jonathan W. Song[2,9,*]

[1]Department of Biomedical Engineering, The Ohio State University, Columbus, OH 43210, USA

[2]Department of Mechanical and Aerospace Engineering, The Ohio State University, Columbus, OH 43210, USA

[3]Interdisciplinary Biophysics Graduate Program, The Ohio State University, Columbus, OH 43210, USA

[4]Department of Physics, The Ohio State University, Columbus, OH 43210, USA

[5]Biomedical Science, College of Medicine, The Ohio State University, Columbus, OH 43210, USA

[6]Department of Radiology, University of Michigan, Ann Arbor, MI 48109, USA

[7]Biointerfaces Institute, University of Michigan, Ann Arbor, MI 48109, USA

[8]Department of Biomedical Engineering, University of Michigan, Ann Arbor, MI 48109, USA

[9]Comprehensive Cancer Center, The Ohio State University, Columbus, OH 43210, USA

***Corresponding authors**

Travis Jones, jones.3318@osu.edu

Jonathan Song, song.1069@osu.edu

Table of Contents

Figure S1: Time-dependence of source concentration. **S3**

Figure S2: Cell viability stain. **S4**

Figure S3: Time of average cytoplasm-to-nuclear ratio (CNR) peak. **S5**

Figure S4: Correlation matrix of experimental parameters and measured cell responses. **S6**

Figure S5: EGF threshold concentration ($EC50_{EGF}$) in multiwell plates. **S7**

Figure S6: Cumulative percentage of activated cells in all conditions in the LDA. **S8**

Figure S7: Representative images of cell coverage in the lateral diffusion assay. **S9**

Figure S8: Dyngo4a inhibits dynamin a vital protein for clathrin-mediated endocytosis**.** **S10**

Table S1: Transport properties of molecules. **S11**

Table S2: Activation delay time by zones in the LDA. **S11**

Table S3: Percentage of activated cells for each condition by zone. **S11**

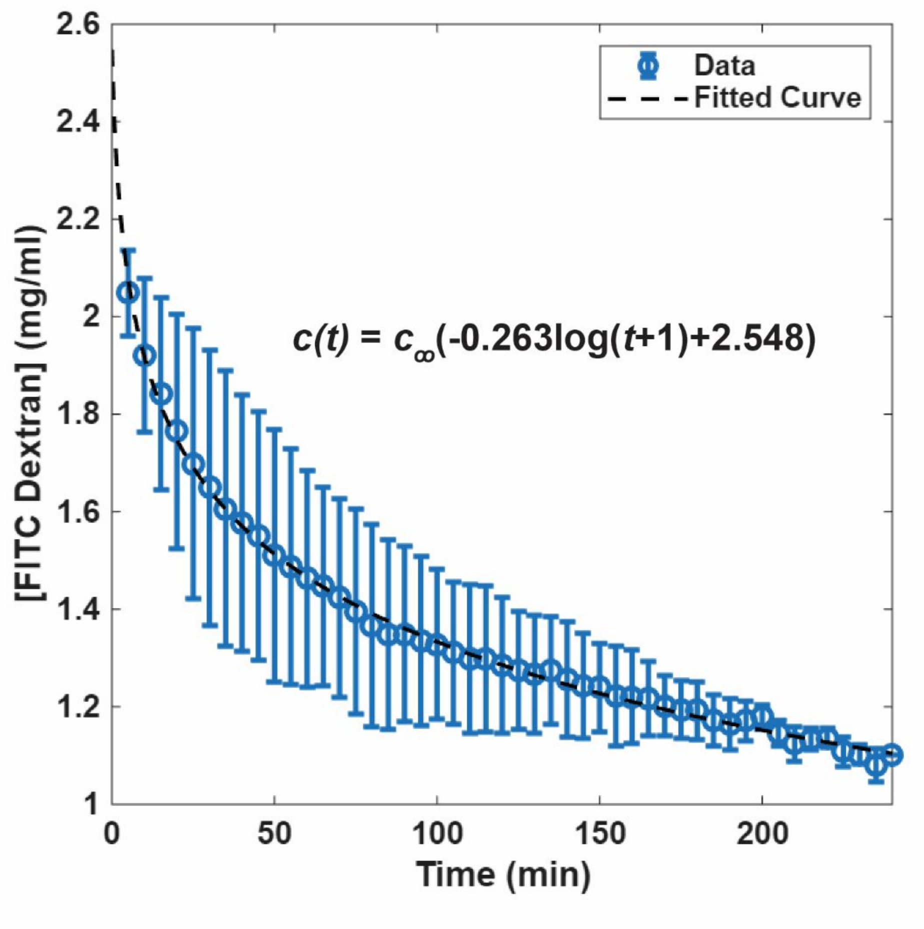

Figure S1: Time-dependence of source concentration. The inlet port concentration was monitored during 10 kDa FITC-dextran diffusion experiments. The 10μl droplet of 10X concentrated solute slowly mixed, approaching 1X concentration at the source over the duration of the experiment. The equation shown was used as a boundary condition for all simulations, where c is concentration of solute, c_∞ is the 1X solute concentration, and t is time in minutes. Data is plotted as mean ± SD (n = 4).

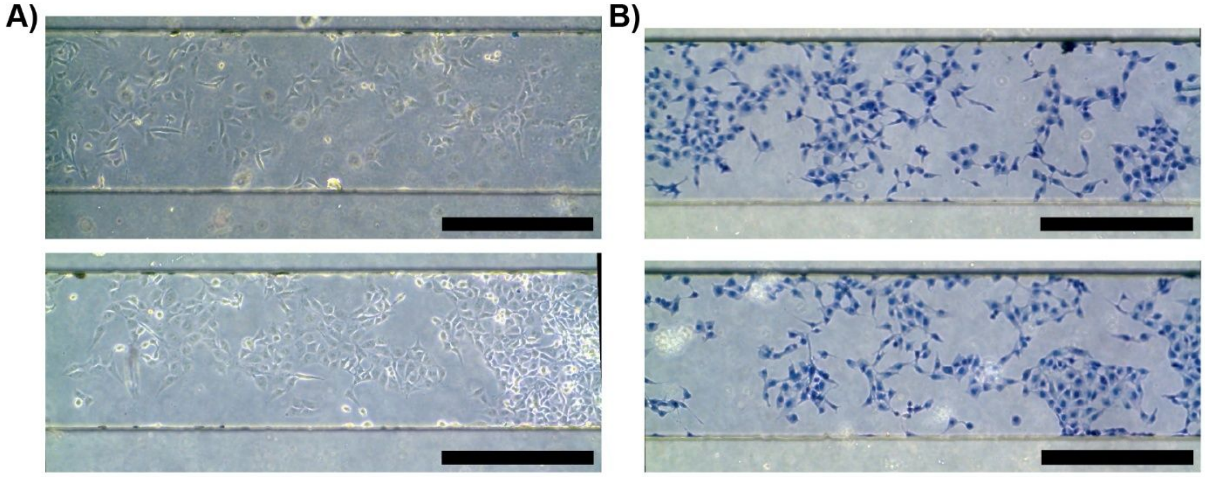

Figure S2: Cell viability stain. **A)** Cells in the lateral diffusion assay maintained at 37°C and 5% CO_2 show almost no cell death after 72 hrs (24 hrs in culture media + 48 hrs in imaging media). **B)** Cells treated at 65°C for 2 minutes show almost complete cell death by evidence of blue dye (Trypan blue) infiltrating the ruptured membranes (Scale bars = 500 µm).

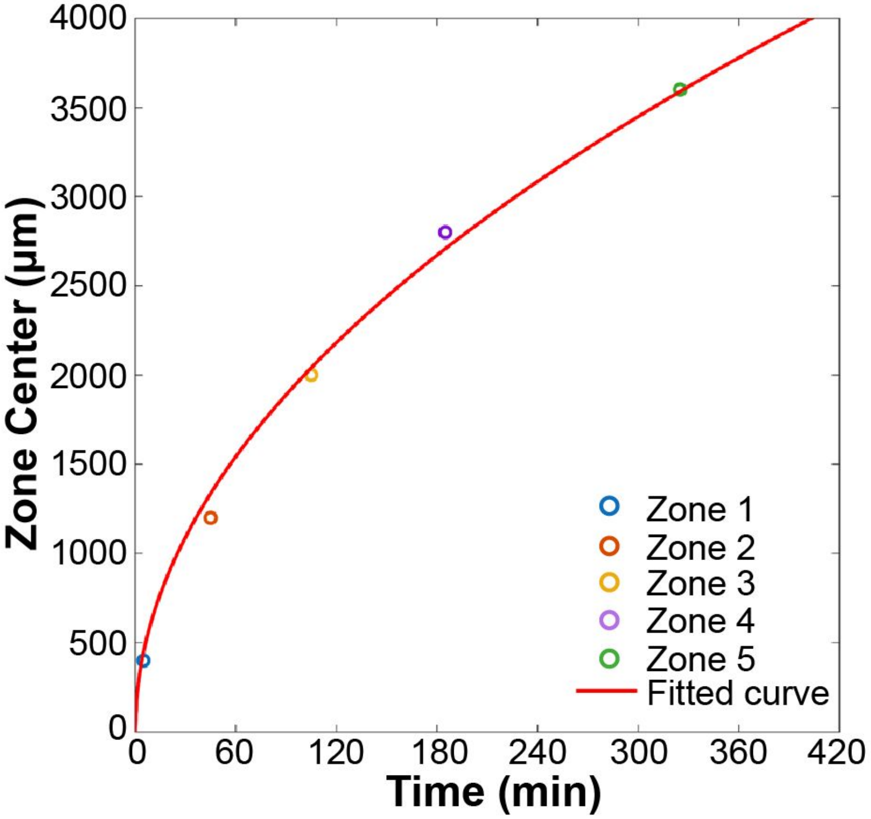

Figure S3: Time of average cytoplasm-to-nuclear ratio (CNR) peak for each zone for 100 ng/ml EGF source concentration (12-hour experiment, n = 1). The center location of each zone is plotted against the time point for the maximum average CNR. The trend follows a square root relationship between location and time ($x \propto \sqrt{t}$) indicating that the cell response is a result of diffusion of ligand.

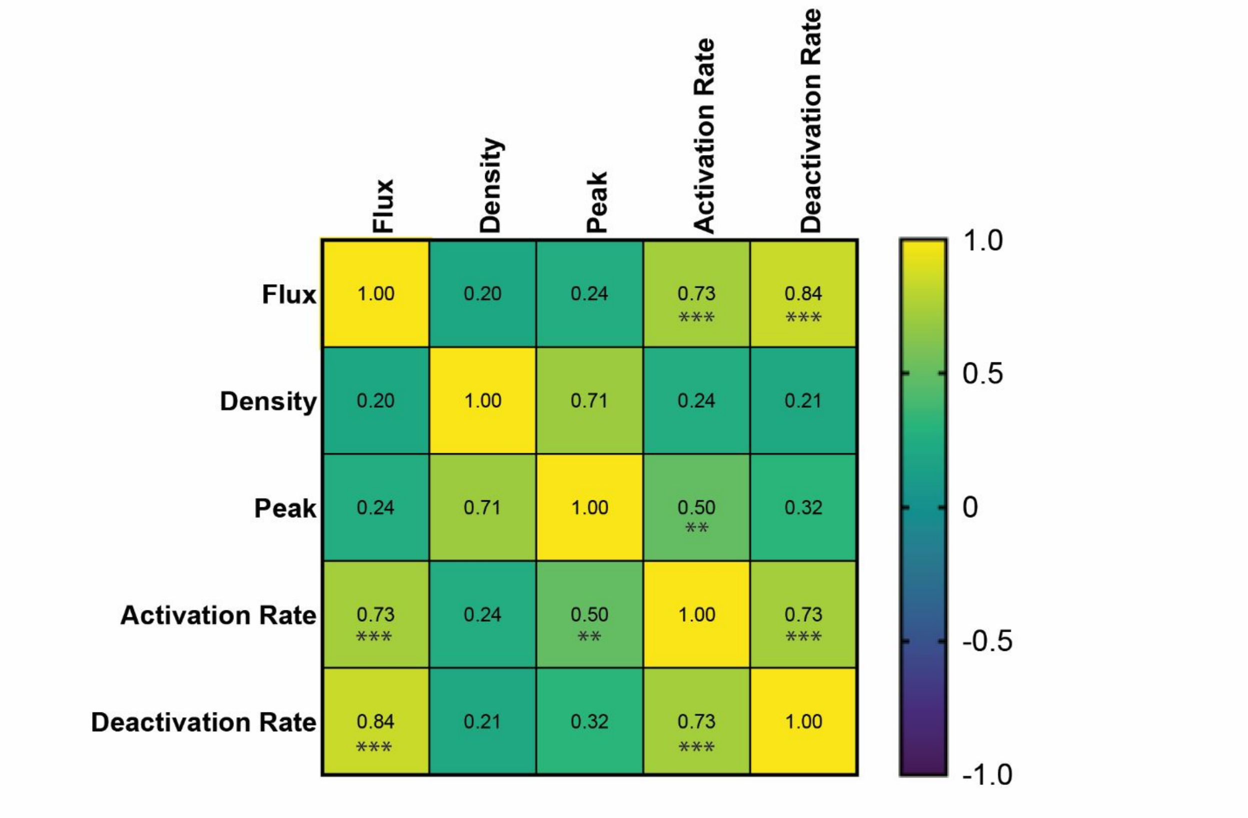

Figure S4: Correlation matrix of experimental parameters and measured cell responses. Experiments are broken up into five zones such that all parameters and measurements are averages for each zone for each experimental condition. Flux is the peak average flux for a zone, density is the average cell density in the zone, peak is the maximum average cytoplasmic-to-nuclear ratio for activating cells, activation rate is the average rate of change of the CNR for 30 minutes prior to peak activation, and deactivation rate is the average rate of change of the CNR for 30 minutes following peak activation. The flux values were $\log_{10}$- transformed prior to analysis. The correlation performed was a Spearman correlation. Peak CNR shows strong positive correlation with cell density. Activation and deactivation rates show strong correlation with EGF flux. (**, $p<0.01$, ***, $p<0.001$).

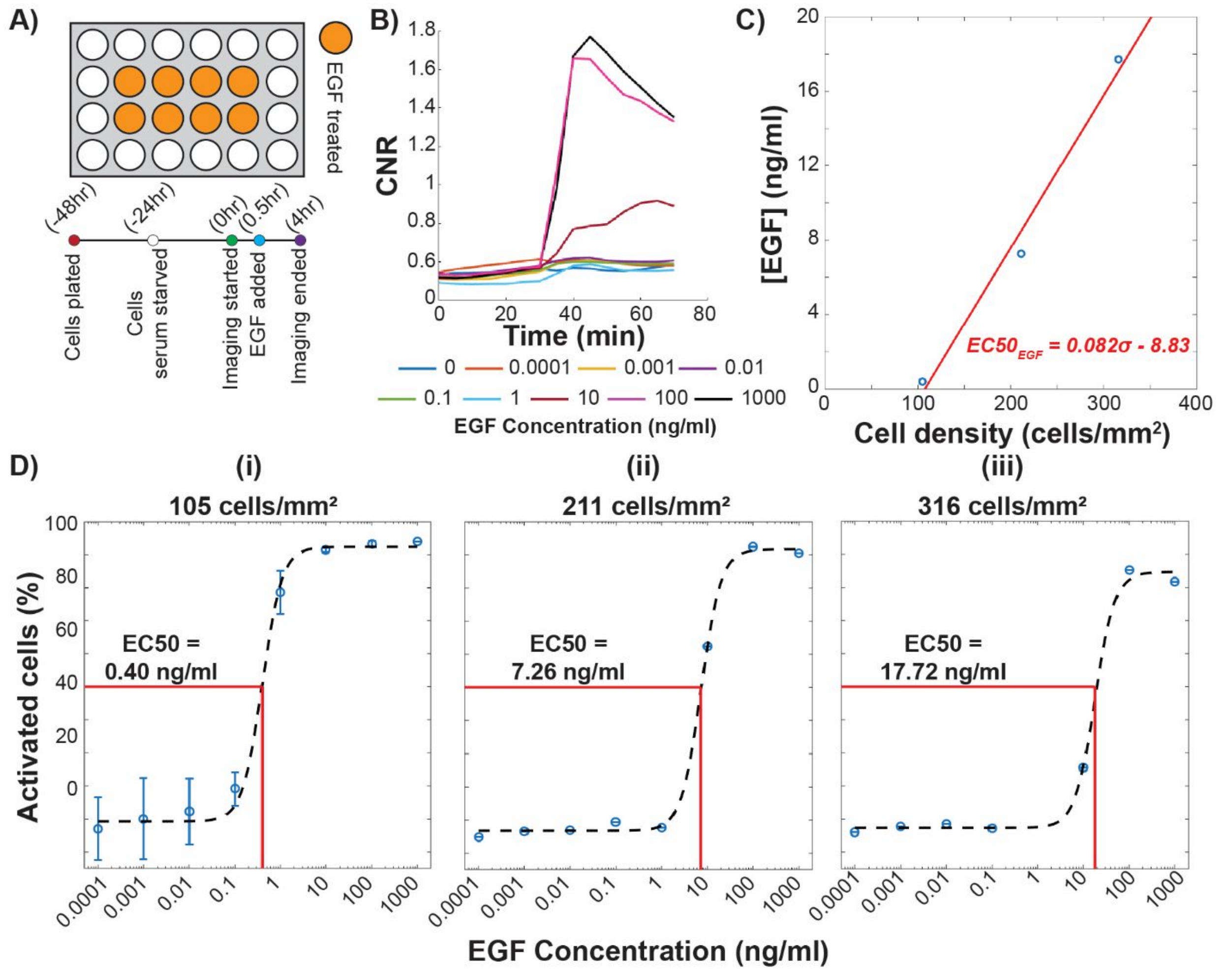

Figure S5: EGF threshold concentration ($EC50_{EGF}$) in multiwell plates. **A)** A 24-well culture plate was used to perform EGF dosage thresholding. Cells were treated with varying concentrations of EGF and imaged over four hours. **B)** The average KTR response for each concentration condition is plotted over time. **C)** $EC50_{EGF}$ as a function of cell density. The solid red line represents a linear fit. $EC50_{EGF}$ is the effective EGF concentration for 50% Akt activation, σ is the cell density. **D)** $EC50_{EGF}$ was found to change with increasing cell density.

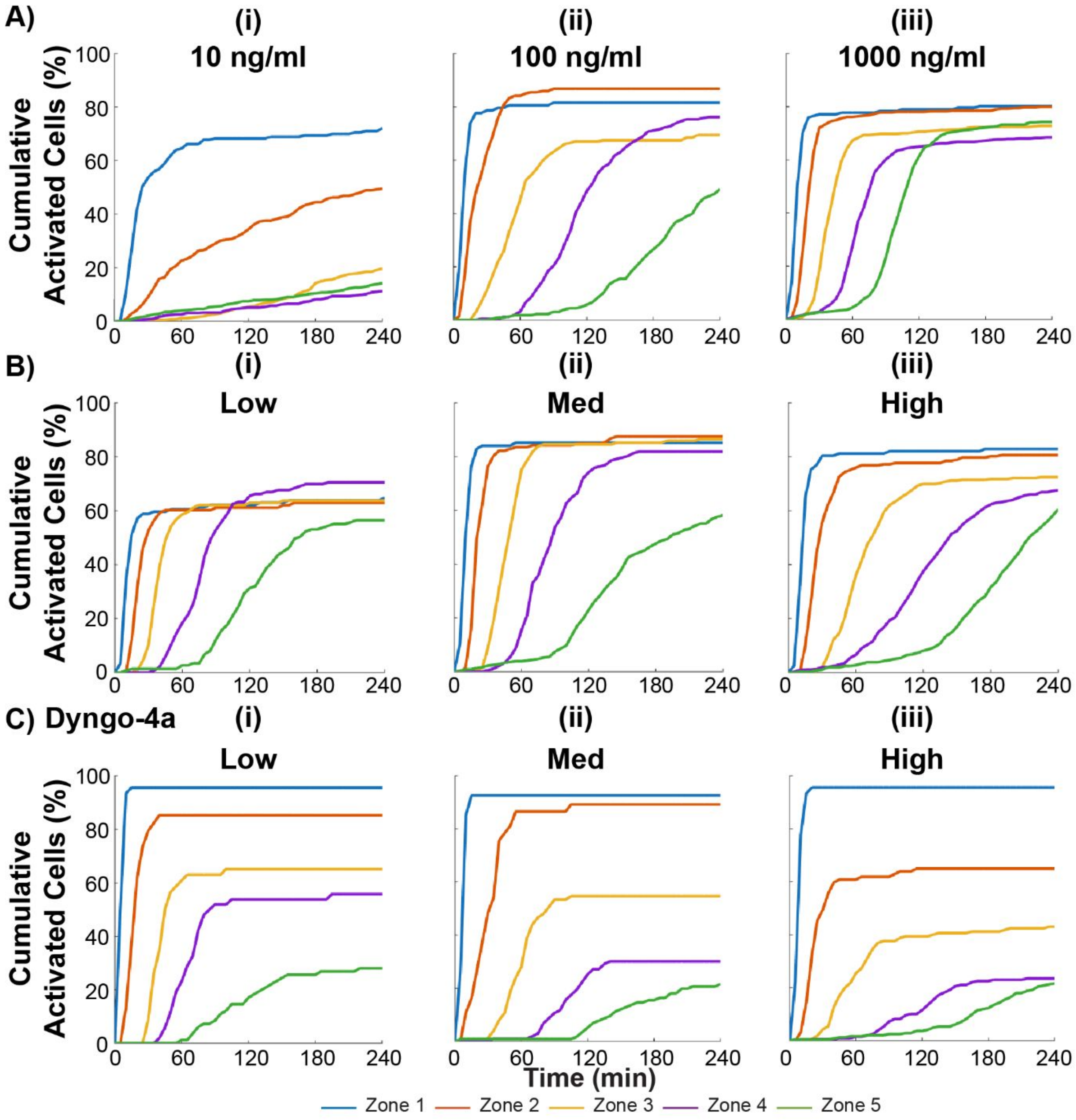

Figure S6: Cumulative percentage of activated cells in all conditions in the LDA. **A)** Varying the source concentration of EGF for **(i)** 10, **(ii)** 100, and **(iii)** 1000 ng/ml. **B)** Varying cell seeding density for **(i)** low, **(ii)** medium, and **(iii)** high densities. **C)** Varying cell seeding density when cells were pretreated with Dyngo-4a for **(i)** low, **(ii)** medium, and **(iii)** high densities. Overall percentages for the corresponding condition and zone are given in **Supplemental Table 3.**

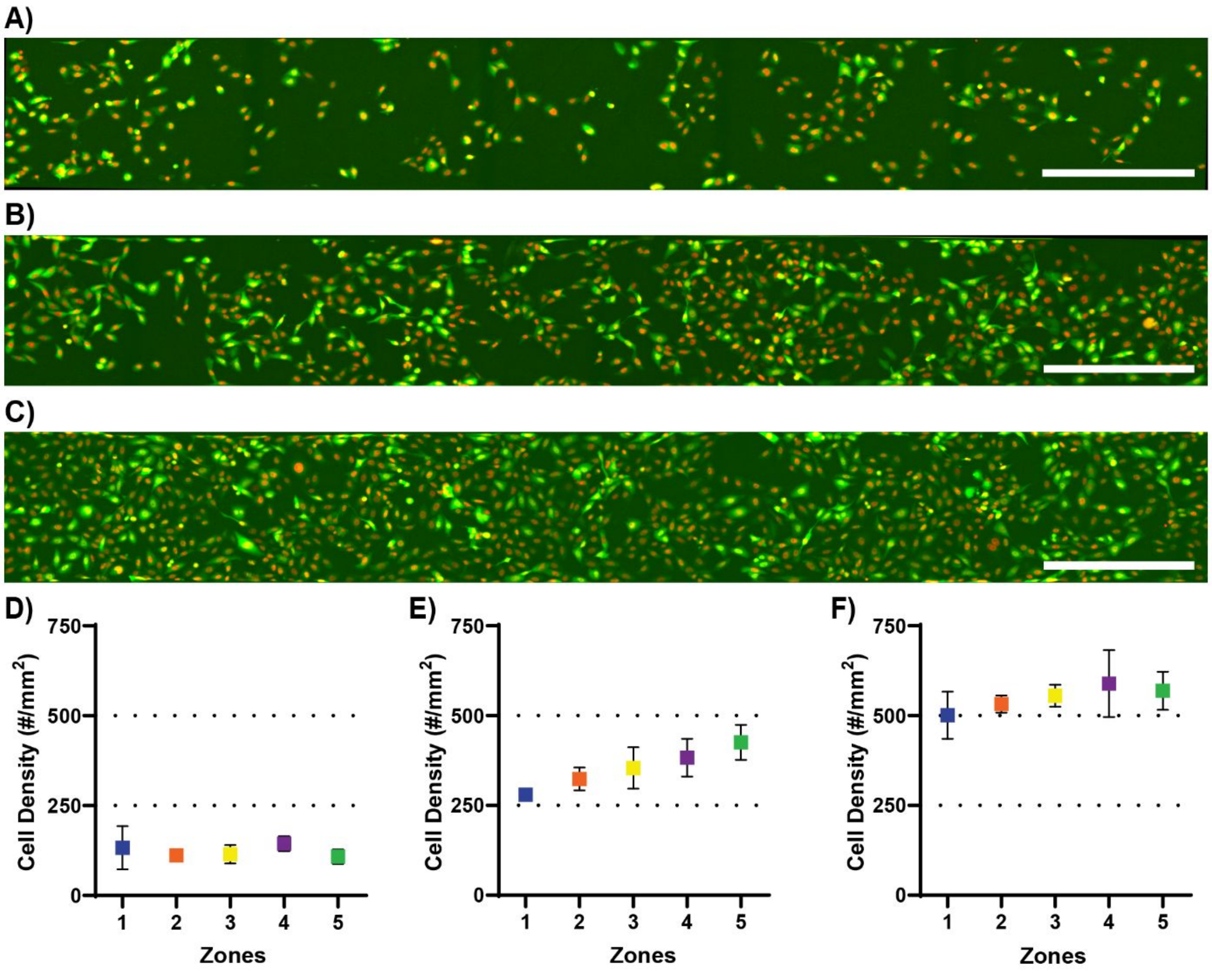

Figure S7: Representative images of cell coverage in the lateral diffusion assay. **A)** Low-density case with cell density of 157.5 cells/mm^2 (compared to 122.2 cells/mm^2 average for condition). **B)** Medium-density case with cell density of 382.5 cells/mm^2 (compared to 352.9 cells/mm^2 average for condition). **C)** High-density case with cell density of 561.5 cells/mm^2 (compared to 547.3 cells/mm^2 average for condition). Brightness and contrast have been adjusted for better visualization of the cells. Red color is the histone H2B tagged with mCherry. Green is the kinase translocation reporter with enhanced green fluorescent protein. Scale bars = 500 µm. Density across the five zones of the channel for **D)** low-density condition, **E)** medium-density condition, and **F)** high-density condition. Data is plotted as mean ± SEM (n = 3).

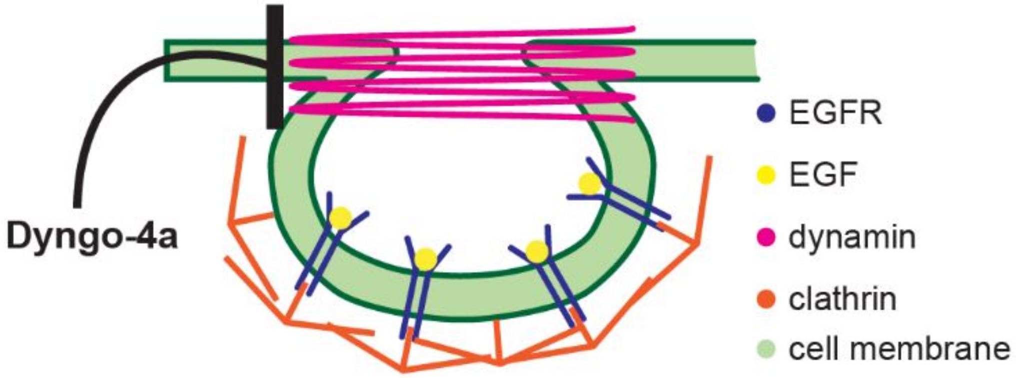

Figure S8: Dyngo4a inhibits dynamin a vital protein for clathrin-mediated endocytosis**.**

Table S1: Transport properties of molecules.

Molecule	Hydrodynamic Radius (nm)	Diffusion Coefficient @ 21°C, 1cP ($\mu m^2/s$)
**10 kDa FITC-dextran**	2.3	93.7
**EGF**	1.4	153.9

Table S2: Activation delay time by zones in the LDA.

Zone	$t_{i,\mathrm{peak}}$ (min)	$t_{i,\mathrm{entry}}$ (min)	$\varphi_{i,\mathrm{delay}}$ (min)	$J_{\mathrm{EGF,peak}}$ ($\mathrm{x}10^{-12}$ kg m^{-2} s^{-1})
**1**	40	0	40	182.4
**2**	80	8	72	14.32
**3**	140	27	113	4.714
**4**	220	55	165	2.183
**5**	360	95	265	1.051

Table S3: Percentage of activated cells for each condition by zone.

Condition	Zone					n
	1	2	3	4	5	
**12h EGF 100 ng/ml**	80.7	84.5	75.3	67.0	73.8	1
**Control**	37.9	21.5	10.0	5.36	6.94	2
**Erlotinib**	5.31	2.27	5.68	4.65	6.61	2
**10 ng/ml EGF**	72.1	49.6	19.7	11.3	14.3	3
**100 ng/ml EGF**	81.6	86.8	69.4	76.2	49.1	2
**1000 ng/ml EGF**	80.2	80.0	72.9	68.5	74.3	2
**Low Density**	64.7	63.0	63.8	70.6	56.5	3
**Medium Density**	85.2	87.5	86.6	81.9	58.2	3
**High Density**	82.9	80.6	72.4	67.5	60.5	3
**Low Density +Dyngo**	95.7	85.3	65.2	55.8	28.0	1
**Medium Density +Dyngo**	92.6	89.2	54.7	30.1	21.6	1
**High Density +Dyngo**	95.6	64.9	43.0	23.4	21.6	1
